# Magnitude, relationship and determinants of attention deficit hyperactivity disorder and depression among University of Gondar undergraduate students, Northwest Ethiopia, 2022: Non-recursive structural equation modeling

**DOI:** 10.1371/journal.pone.0291137

**Published:** 2023-10-05

**Authors:** Werkneh Melkie Tilahun, Haileab Fekadu Wolde, Zenebe Abebe Gebreegziabher, Wondwosen Abey Abebaw, Mulat Belay Simegn, Asefa Adimasu Tadesse

**Affiliations:** 1 Department of Public Health, College of Medicine and Health Science, Debre Markos University, Debre Markos, Ethiopia; 2 Department of Epidemiology and Biostatistics, Institute of Public Health, College of Medicine and Health Sciences, University of Gondar, Gondar, Ethiopia; 3 Department of Epidemiology and Biostatistics, School of Public Health, Debre Birhan University, Debre Birhan, Ethiopia; 4 Department of Epidemiology and Biostatistics, School of Public Health, College of Medicine and Health Sciences, Woldia University, Woldia, Ethiopia; Ambo University, ETHIOPIA

## Abstract

**Background:**

Up to 80% of adults with attention deficit hyperactivity disorder (ADHD) may have a concomitant psychiatric disorder. ADHD and depression, combined with the demands of University life, can pose serious challenges for students. However, there are limited studies conducted on this topic in our country. Therefore, the current study assessed the magnitude, relationship, and determinants of ADHD and depressive symptoms among students at the University of Gondar.

**Method:**

An institution-based cross-sectional study was employed among 1514 undergraduate students at the University of Gondar from June 1–20, 2022. A stratified, simple random sampling technique was applied. Structural equation modeling was employed. The degree of relationship was interpreted based on the adjusted regression coefficient with 95% confidence interval (CI) and p-value (<0.05).

**Result:**

In our study, 37.6% [CI: 35.2–40.1%] and 20.01% [CI: 18.1–22.1%] of the participants had depressive and ADHD symptoms, respectively. Chronic illness [β = 0.19, CI; 0.09, 0.30], alcohol use [β = 0.10, CI; 0.03, 0.17], social support [β = -0.23, CI; -0.29, -0.17], problematic internet use (PIU) [β = 0.23, CI; 0.18, 0.27], and insomnia [β = 0.24, CI; 0.17, 0.30] had a direct relationship with depressive symptoms. Mother education level [β = -0.09, CI; -0.13, -0.06], birth order [β = -0.09, CI; -0.11, -0.07], chat chewing [β = 0.18, CI; 0.06, 0.30], and depressive symptoms [β = 0.73, CI; 0.62, 0.86] had a direct relationship with ADHD. Chronic illness [β = 0.14, CI; 0.06, 0.22], PIU [β = 0.16, CI; 0.13, 0.21], social support [β = -0.16, CI; -0.22, -0.12], and insomnia [β = 0.17, CI; 0.13, 0.23] had an indirect effect on ADHD.

**Conclusion and recommendation:**

The prevalence of ADHD and depressive symptoms was high. Chronic disease, alcohol use, social support, PIU, and insomnia had a direct effect on depressive symptoms and an indirect effect on ADHD. Mother education, birth order, chat chewing, and depressive symptoms had a direct effect on ADHD. Our study provided useful clues for clinical treatment and school-based interventions that aim to improve college students’ mental well-being. It is better to design school-based intervention and prevention programs to achieve timely diagnosis and treatment of these disorders among university students.

## Introduction

Attention deficit hyperactivity disorder (ADHD) is one of the most common mental disorders explained by three main symptoms, such as inattention, hyperactivity, and impulsivity [1,2]. It is a prevalent neurodevelopmental disorder that can be diagnosed during adulthood [3] and affects people of all ages [4]. The overall prevalence rate of diagnosed ADHD increased by 10.1 times in adults (>18 years) between 2008 and 2018 [5]. Among college students, the global prevalence of self-reported ADHD between 2014 and 2018 was 15.9% [6], with high comorbidity among this population [7]. In Africa, according to a study conducted among Kenyan undergraduate students, the prevalence rate of self-reported ADHD symptoms was 21.8% [8]. Another web-based cross-sectional study conducted among medical students in Cameroon revealed the prevalence of self-reported ADHD symptoms at 24.4% [9].

Despite the strong contribution of genetics, there is no single cause of ADHD. Its cause is thought to be a combination of genetic, biological, and environmental factors [10]. Evidence showed a 2- to 8- fold increased risk of ADHD among children of parents and siblings affected by ADHD [11]. Environmental factors such as maternal-related prenatal risks, pregnancy, and birth complications, as well as external agents like infections, have their own contributions [12].

ADHD has an impact in all spheres of life [13,14]. University students with ADHD are at greater risk of impaired educational functioning or poor academic performance [15], lower scores on total well-being, environmental mastery, impaired personal growth, loss of purpose in life, and difficulties in their organizational and goal-oriented competencies [16]. In addition, it enhances the risk of depression later in life [17].

ADHD is strongly associated with depression and mood disorders during adulthood [18]. Even if the link between ADHD and depressive symptoms is not yet fully understood [19], studies show the prevalence of comorbid depression among adults diagnosed with ADHD is more than nine times higher [20] and ranges between 17.3% [21] and 60.7% [22]; also, the prevalence of ADHD among adults with depression ranges between 7% [23] and 22.1% [24]. Higher ADHD symptoms may cause a worse and earlier clinical presentation of depression, higher depression-related impairment, increased recurrence, persistence of depressive episodes and symptoms, risk of self-harm or suicide attempt, hospitalization, and receiving non-first-line antidepressant medication [25]. Depression and ADHD coexistence is more severe, impairing and complicating treatment of both disorders [26]. Psychiatric comorbidity in general is a key predictor of more treatment-resistant depression [27]. Thus, ADHD is expected to interfere with psychosocial treatments, medication adherence, and treatment outcomes [28].

Depression is also a common mental disorder and the leading cause of disability [29]. Among college students, severe depression escalated from 9.4% to 21.1% from 2013 to 2018, and the rate of moderate to severe depression raised from 23.2% to 41.1% between 2007 and 2018 [30]. Depression causes significant impairment across numerous areas of functioning [31]. It causes physical impairments of a similar magnitude as chronic diseases such as diabetes and cancer, with elevated morbidity and mortality [32]. Even individuals who report mild impairment are at greater risk of hospitalization, suicide, and unemployment than individuals without depression [33].

Despite the fact that the diagnosis of ADHD among adults is growing four times faster compared to children [34], fewer than 20% of adults with ADHD are currently diagnosed and/or treated by psychiatrists [35]. University life is a critical stage for students since they are exposed to new situations such as independence, changes in peer groups, new social situations, worry about increased responsibility and finances, and increased accessibility to alcohol and drugs [36]. This may lead to negative psychological effects like depression [37]. There is also a marked increase in the prevalence of ADHD among University students [38], but our knowledge and understanding are relatively limited [39]. Undergraduate students reported significantly higher rates of mental health issues than graduate students [40].

Previous studies among adults or university students revealed that socio-demographic factors like being female [41], younger age [8], low parental education [42,43], and academic failure [44] were risk factors for symptoms of ADHD. Clinical factors like being the firstborn, parental history of psychiatric disorders [7], diagnosis of depression [7,9,41,45], history of brain injury, and histories of chronic disease [9] were risk factors for ADHD. And behavioral factors like cigarette smoking, alcohol drinking, and other drug use like cannabis [44,46,47] were found to be independent predictors of symptoms of ADHD.

And also socio-demographic factors like being female [48–50], age [51–53], prior urban residence [50,54], inadequate monthly allowance [55], poor family economic level [56], higher academic year compared to first year [48,53,57], health departments [54], worrying about academic performance [52,53], reduced number of sleeping hours during the night, higher studying hours positively, poor and moderate social support [49,58], and a lack of a stable partner [51] were associated with an increased risk of depression. Behavioral factors like alcohol drinking [48,55], lack of adequate physical exercise [54,56], cigarette Smoking [48,51], and problematic internet use [51,56] were related to depression. And also, clinical factors like a history of chronic illness [49], insomnia [51,52,57], and a history of stressful life events [59] increase the risk of depression.

Although previous studies revealed a higher mental health problem among undergraduate students, the magnitude of ADHD and its relationship with other psychiatric disorders like depression have not previously been explored in our country. And also, ADHD is an emerging mental health problem and a neglected health issue in Africa [60]. Symptoms of ADHD and depression combined with the demanding nature of University life, such as increased academic, organizational, and social demands, may pose a serious challenge for students. Which in turn may affect measures of success in the higher education system. Furthermore, early recognition and treatment of ADHD have the potential to alter the trajectory of other comorbid psychiatric disorders.

Although SEM has been applied in numerous fields, epidemiology and medical research have not yet made considerable use of it. It is a flexible framework for developing and evaluating complex relationships between numerous variables that enables researchers to use empirical models to assess the viability of an existing theory. The capacity to control measurement error is one of its biggest advantages. However, it is one of the greatest limitations of most studies. [61].

Hence, exploring the magnitude of ADHD and depression symptoms as well as their relationship among undergraduate university students would shed light on the phenomenology of the problems among young adults seeking higher education and enable targeted early diagnosis and therapeutic interventions for vulnerable students. Given the coexisting mental illness of ADHD, evidence on the prevalence and relationship of adult ADHD with depression among young adult college students is vital to designing early diagnosis and intervention to inform appropriate management decisions in order to improve control measures and promote quality and safe mental health practices among students. In addition, this study provides updated and baseline information and methodological advancements for future researchers to further refine and prioritize specific areas of future research in the areas of ADHD, depression, and their relationship. Thus, this study examined the magnitude, a bidirectional relationship, and determinants of ADHD and depressive symptoms among students at the University of Gondar using non-recursive structural equation modeling.

## Methods and materials

### Study setting, design, and period

An institution-based cross-sectional study design was employed from June 1–20, 2022, at all five campuses (Science Amba, Atse Tewodros, Fasiledus, Maraki, and Tseda) of the University of Gondar. A total of 6 colleges (College of Medicine and Health Sciences, Business and Economics, Social Science and Humanities, Natural and Computational Science, Agriculture and Environmental Science and Veterinary Medicine and Animal Science), 2 institutes, (Institute of Technology and Biotechnology), two faculties (Faculties of Informatics and Education) and one school (School of Law) are found in all campuses, offering 56 undergraduate and 64 postgraduate programs in regular, extension, distance, and summer programs [62]. There were about 9905 students enrolled in the undergraduate regular program during the data collection period.

### Population

All regular undergraduate students at the University of Gondar were the source population, and all regular undergraduate students registered for the 2022 academic year and available at the University of Gondar during the data collection period were the study population.

### Eligibility criteria

In this study, all regular undergraduate students who registered at the University of Gondar for the academic year 2022 were included. However, students with medically confirmed thyroid disease and below the age of 18 were excluded from the study.

### Sample size determination

Structural equation modeling (SEM) is a large-sample technique, and the sample size required is dependent on the complexity of the model. As a general rule of thumb, the minimum sample size should be no less than 5–20 times the number of parameters to be estimated [63]. Thus, in our case, we used the N:q ratio of 10:1 to calculate the number of observations required to estimate the parameters of a hypothesized relationship constructed among variables based on a literature review and the plausibility of relationships. Free parameters to be estimated can be calculated by adding the number of paths except those paths fixed to 1, the number of variances of exogenous variables (both latent and observed), the number of covariances and disturbance (error) terms.

Accordingly, there were 34 observed endogenous variables (6, 9, 9, 7 and 3 for ADHD, depression, problematic internet use (PIU), insomnia, and social support respectively). Therefore, 29 path coefficients were needed since five of them were fixed to 1, in order to give the latent variable measurement scale. Two disturbance terms, two paths between ADHD and depression, and one covariance between disturbance terms and there are 34 error terms for those observed endogenous variables. There are also 26 observed exogenous variables (9 for depression only, 3 for ADHD only, and 14 for both) and three latent exogenous variables. Therefore, the total number of free parameters to be estimated is equal to

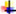
 34*2–5 = 63 path coefficients and error terms for latent variables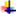 3*2 = 6 path coefficients and variance for exogenous variables for ADHD only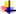 12*2 = 24 path coefficients and variance for exogenous variables for depression only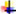 14*3 = 42 path coefficients and variance for common factors for both outcomes.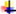 **2 + 2+1 = 5 for** the feedback loop, their disturbances, and covariance.

The pilot study suggests **seven** significant covariances between error terms. The total number of parameters to be estimated was **147.** Therefore, 147*10 = 1470. A non-response rate of **5%** was considered. Then the final calculated sample size was **1544**. Even though the sampling technique is stratified sampling and needs a design effect, available evidence declares that the design effect for the mean of a proportionate sample is no greater than 1. Thus, proportionate stratification cannot lead to a loss in precision but generally leads to some gain in precision [64].

### Sampling procedure

**Fig 1:**
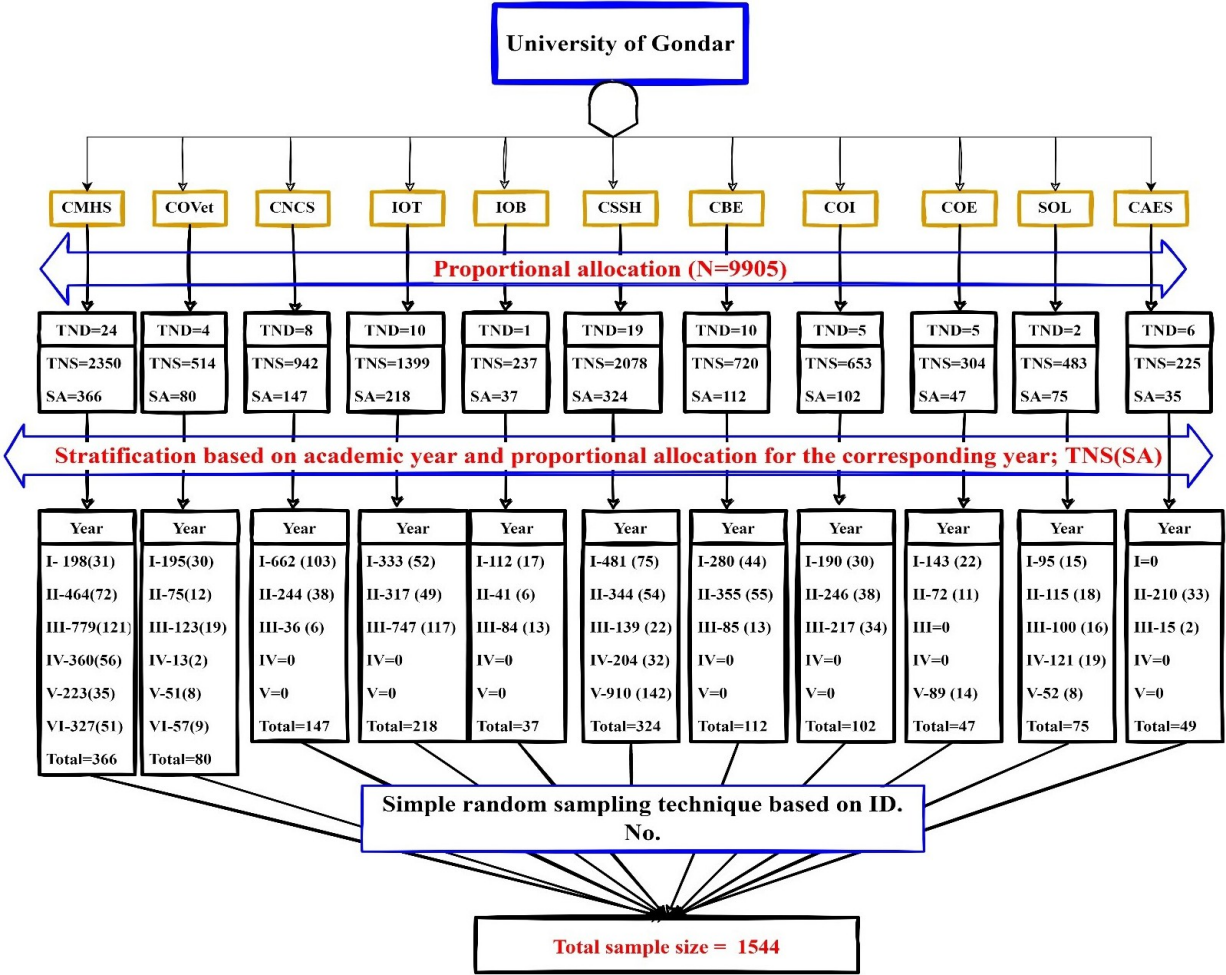
Schematic presentation of the sampling procedure applied to select study participants, UoG, Northwest Ethiopia, 2022. Where N = total number of students available during data collection. CMHS- college of medicine and health science, COVet- college of veterinary medicine; CNCS- College of natural and computational science; IOT-institute of technology; IOB- institute of biotechnology; CSSH- College Of Social Science And Humanities; CBE-College Of Business And Economics; COI- College Of Informatics; COE- College Of Education; SOL-School Of Law; CAES- College Of Agriculture And Environmental Science; TND- Total Number Of Departments; TNS- Total Number Of Students; SA-Sample allocated and ID.no- Identification number.

Stratified simple random sampling was applied to select the study participants. The number of students in each campus and college/institute/facilities/school was determined, and then the total sample size was proportionally allocated for each campus and college/institute/faculties/school. Then stratification based on the academic year under each college/institute/facilities/school was employed. Then the proportionally allocated sample size for each college, institute, facility or school again proportionally allocated for each academic year. Finally, the study participants were taken randomly from each year by computer generated random sampling based on their ID No. **(Fig 1).**

### Variables of the study

**Outcome variables.** ADHD and depression (latent endogenous variables)

**Independent variables. Latent exogenous variables** (insomnia, problematic internet use and social support)

Observed exogenous variables such as;

**Socio demographic factors:** sex, age, monthly allowance, maternal education, father education, prior residence, self-rated family economic level and presence of partner.

**Health related factors:** history of chronic disease, history of head injury, history of parental psychiatric illness, birth order and history of stressful life events.

**Behavioral factors:** history of alcohol use, current alcohol use, cigarette smoking, chat chewing, cannabis use, and physical exercise

**Academic related factors:** year of study, number of studying hours per day, number of sleeping hours per day and worry about academic performance.

### Measurement and data collection tool

The tool incorporated questions regarding socio-demographic, clinical, academic, and behavior-related factors, as well as about ADHD, depression, and PIU.

**ADHD symptom:** it was assessed using the six-item Adult Self-Report Scale-V1.1 (ASRS-V1.1), a short screener from the World Health Organization Composite International Diagnostic Interview. All questions have five choices: never, rarely, sometimes, often, and Very often. The scoring is binary: either 0 or 1 for determining whether the participant had the symptom. For questions 1–3 the scoring is: Never and rarely– 0, while Sometimes, Often and Very often—1. For questions 4–6, the scoring is: Never, Rarely and Sometimes– 0, Often and Very often—1. Having a score of four or more “1”s with the presence of symptoms in ≥2 settings over the past 6 months making the diagnosis [65].

**Depressive symptom:** it was determined by the Patient Health Questionnaire-9 (PHQ-9) tool. It has nine questions that ask about the frequency of the occurrence of symptoms. All can be scored from zero to three as not at all, several days, more than half the days and nearly every day, respectively. There can be a minimum score of 0 and a maximum of 27. In this study, PHQ-9 scores ≥ 10 were considered as positive for depressive symptoms, and their severity was defined as mild: scores of 5–9; moderate: scores of 10–14; moderately severe: scores of 15–19; and severe: scores of 20–27 [66].

**Insomnia:** The measurement was made using the insomnia severity index, which has seven questions that asked about the last two weeks insomnia problems. It has 5 alternatives, which can be scored from 0 to 4, and higher sores indicate higher risk of insomnia [67].

**Social support:** was measured using the Oslo Social Support Scale (OSSS-3). It consists of three items. The first question has four alternatives ranging from 1–4, and the rest has five alternatives ranging from 1–5. Higher OSSS-3 scores indicate a better social support [68].

**Problematic internet use:** it was measured using the Problematic Internet Use Questionnaire-9 (PIUQ-9); it is a brief version of the long PIU-18 items. It has nine questions with a possible five responses. 1 “Never”, 2 “Rarely”, 3 “Sometimes”, 4 “Often”, and 5 “Always/almost always” available elsewhere [69,70]. Higher scores show a high problem with internet use.

**Ever-smoking** was assessed by asking students whether they had ever smoked cigarettes during their lifetime [71]. The responses were “Yes” or “No”.

**Physical exercise:** Exercising or doing any kind of sports activity, including walking for at least 20 minutes per day; and the responses were “Yes” or “No” [72].

**History of alcohol use:** students who answered “Yes” to the question “have you ever drunk at least one of the alcoholic beverages (beer, wine, whiskey, Areki, Tela, Tej, etc.) for nonmedical purposes?” were considered ever-alcohol users [73].

**Current alcohol use:** students who answered “Yes” to the question “have you ever drunk at least one of the alcoholic beverages (beer, wine, whiskey, Areki, Tela, Tej, etc.) for nonmedical purposes in the last three months?” were considered current alcohol users [73,74].

**Chat chewing** was assessed by asking a single question: “Have you ever chewed chat during your lifetime?” and possible responses were “Yes” or “No” [73].

**Cannabis use** was assessed by asking students “whether they have ever used cannabis,” and possible responses were “Yes” or “No” [75].

**The history of parental psychiatric illness** was assessed using one question: "Do your parents have a history of any medically confirmed psychiatric disorders? (It can be any type). Possible responses were “no”, “father”, “mother”, and “both”.

#### Data collection procedure

Data was collected using a self-administered, standardized questionnaire. Data collection was done by four trained data collectors from a health background (public health) under the direct supervision of the researchers. Once a student’s identification number was selected using Excel, data collectors found the corresponding participants. After the aim, benefits, and harms of the study were explained and students agreed to participate, the questionnaire was administered. The Completeness and consistency of the responses were immediately checked by the data collector and the principal investigators, respectively. All the processes were under the direct supervision of the principal investigators.

### Data quality assurance and management

To maintain the quality of the data, a pilot study was conducted prior to actual data collection. The face validity of the instrument was checked by senior and active psychiatrists. The questionnaire that was originally developed in English was translated into the local language, Amharic, which is widely spoken and the official working language of Ethiopia. And then re-translated into English by another person to ensure proper translation, consistency, and conceptualization of terminologies in local contexts. Data collectors were trained about the content, issues of confidentiality, ethical conduct, and data collection techniques for two days. During the data collection process, the researchers were always in timely and close contact with the collectors to resolve any questions or ambiguities about the procedures and provide guidance where necessary. The Collected data were immediately checked for completeness and consistency, and early correctional measures were taken. During the data entry time, consistency, any entry errors, missing values, and outliers were checked manually by referring the questionnaire back.

#### Reliability and validity of tools

The reliability and validity of the tools for the above-mentioned latent variables have not been validated for students in Ethiopia. Tools validated in other contexts may not be reliable and valid to ensure that the instrument used is measuring what it is supposed to measure due to different socioeconomic, cultural, and lifestyle differences. Therefore, to validate the tools, an external, participatory pilot study was conducted among 156 Debre Markos University students. Then confirmatory factor analysis was done to further check the factor loadings with their corresponding p-values, internal consistency, construct validity, and statistical validity.

Internal consistency was checked by composite reliability (CR). Thus, the composite reliability of all constructs ranges between 0.64 and 0.9. Therefore, all constructs met the acceptable level of 0.60 [76]. Construct validity (convergent validity) was checked by average variance extracted (AVE) and CR. Accordingly, AVE ranges between 0.38 and 0.49. Though AVE was below 0.5, the convergent validity of the tool was maintained. Since AVE is a more conservative estimate of the validity of the measurement model [76,77], on the basis of CR alone, we can conclude that the convergent validity of the construct is adequate. In our case, all items standardized load was significant on their corresponding factor (P < 0.001), and composite reliability was above 0.6. Discriminant validity was assessed by comparing the square root of AVE and the correlation between factors. Thus, discriminant validity was satisfied since all factor correlations were less than the square root of AVE for each factor **(S1 and S2 Tables).**

### Omitted variable bias

Omitted variable bias (OVB) is a condition that violates the exogeneity assumption and occurs when a specified regression model excludes a third variable that can affect both the independent and dependent variables in the causal pathway [78]. In our case, depressive and ADHD symptoms have a bidirectional relationship. Therefore, if a variable outside the model that can affect depressive or ADHD symptoms is missed, OVB will be committed. Therefore, to assure the absence of this problem, a regression specification-error test (RESET), which tests the model to ensure that the omitted variables are not causing model misspecification, was conducted, and the result indicated the absence of the omitted variable problems in our study since P-Values for both outcome variables ADHD and depression symptoms were 0.47 and 0.67, which is > 0.05. Hence

**Ho:** The model has no omitted variables.

### Data processing and analysis

The data was entered into Epi-Data Version 4.61. Amos SPSS version 21 and STATA version 16 software were used for analysis. Assumptions like multivariate normality, sampling adequacy, sphericity, missing data, and outliers were checked. Descriptive analysis was conducted by generating a frequency table, percentage, mean/median, and graph. Then SEM was employed to test and estimate complex relationships between variables. The degree of relationship was interpreted based on the adjusted regression coefficients with a 95% confidence interval (CI) and a corresponding p-value < 0.05. Five logical steps were followed in the model building process: model specification, identification, parameter estimation, model evaluation, and modification [79].

**1. Model specification is** the first step by which the researcher identifies the concept to define the hypothesized relationships among the variables. It involved determining every relationship and parameter in the model for the interest of the researcher [80]. In our case,

**Common factors:** year of study, age, sex, cannabis use, chat chewing, current alcohol use, history of head injury, history of smoking, history of alcohol use, history of academic failure, history of family psychiatric illness, prior residence, history of chronic disease, and physical exercise

**Factors related to depression only:** worry about academic performance, monthly allowance, history of stressful life events, marital status, having a closely related friend, average sleeping and studying hours, self-rated family economic level, department type, social support, problematic internet use, and insomnia.

**Fig 2:**
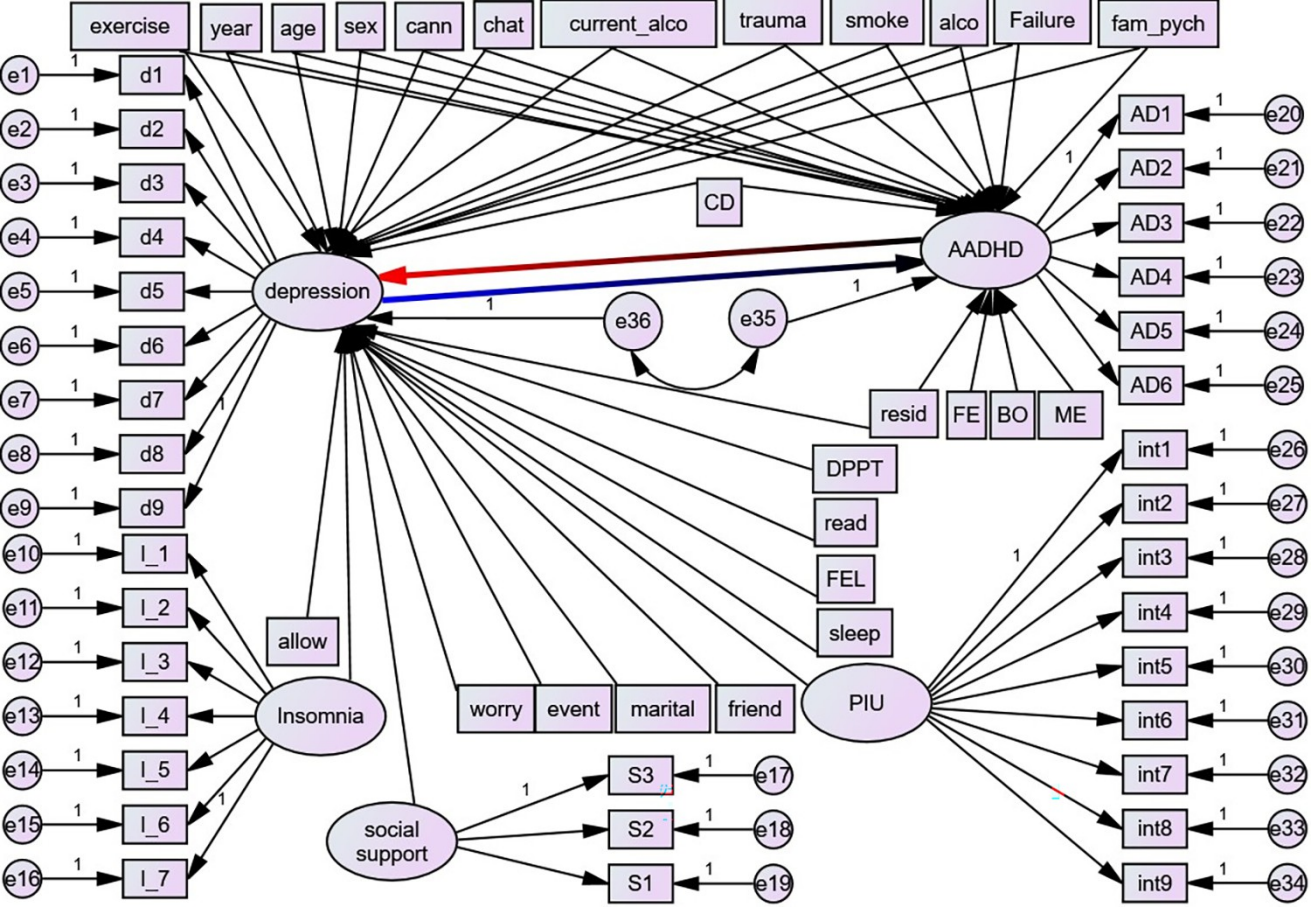
Hypothesized structural model, UoG, Northwest Ethiopia, 2022. Circles indicate latent variables or error terms or disturbances, rectangles indicate observed variables, single arrows indicate factor loadings or regression coefficients, and double arrows indicate the covariance between latent variables; AADHD = Adult Attention Deficit Hyperactivity Disorder, d1-d9 –depression items, AD1-AD6 = ADHD items, S1-S3 = items for Social Support, I_1—I_7- Insomnia Items, exercise = Physical exercise, year = year of study, cann = cannabis, current_alco = current alcohol, alco = alcohol use, failure = history of academic failure, fam_pych = history of family psychiatric illness, allow- Monthly Allowance, worry = worry about academic performance. Event = history of stressful life event, FEL = family economic level, read and sleep = reading and sleeping hours per day, FE = father education level, ME = mother education level, BO = birth order, PIU = Problematic Internet Use, int1-9 = items for Problematic Internet Use, DPTT = department type and CD = Chronic Disease.

**Factors related to ADHD only:** mother and father’s education level and birth order (**Fig 2**).

**2. Model identification**

It is critical to check if the model was identified. It involves the study of conditions to obtain a single, unique solution for each free parameter specified in the model from the observed data. There are three types of model identification. Under-identified: if a single, unique value cannot be obtained from the observed data for one or more free parameters, just-identified: if each free parameter value can be obtained through one and only one manipulation of the observed data, over-identified: if a value for one or more free parameters can be obtained in multiple ways from the observed data, If the model is over-identified: the degree of freedom will be positive [81]. Model coefficients can only be estimated in the just-identified or over-identified model [80].

Identification of non-recursive structural models is more concerning, complicated, and not always identified. To determine whether a non-recursive model meets certain requirements for identification, there are rules only necessary for identification (order condition), which means that satisfying them does not guarantee identification. If a non-recursive model satisfies a sufficient condition (rank condition), however, then it is identified [81]. The third important thing for the identification of non-recursive SEM is the presence of instrumental variables.

**Order condition:** It is a counting rule applied to each endogenous variable in a non-recursive model. If it is not satisfied, the equation for that endogenous variable is under-identified. It can be satisfied when the number of excluded variables for each endogenous variable equals or exceeds the total number of endogenous variables minus 1, i.e., K ≥ M-1, where K is the number of excluded variables and M is the number of endogenous variables in a loop [81].

**Rank condition:** evaluation of the rank condition is sufficient to know identifications of non-recursive models. It is the presence of a unique pattern of direct effects on each variable in a feedback loop from variables outside the loop [81]. In our case, we had two endogenous variables (M) in the loop: ADHD and depression. There were 12 variables that could only affect depression. Therefore, the number of excluded variables for ADHD was 12 = K, 12 > 2–1 and 12 >1 **it is identified.** There were three (3) variables that could only affect ADHD. Therefore, the number of excluded variables for depression was 3 = K → 3 > 2–1 → 3 >1 **it is also identified.** The order condition for our model is already satisfied, but it is not sufficient to say the model is identified. Therefore, the rank condition for each equation (depression and ADHD) should be checked. Evaluation of the rank condition was done using the system matrix. We had first separately put the complete equation of the simultaneous paths. The equation for depression was;

depression(Y1)=x1year+x2age+x3sex+x4cannabis+x5chat+x6currentalcoholuse+x7headtrauma+x8smoking+x9everalcoholuse+x10exercise+x11readinghours+x12academicfailure+x13parentalpsychiatricillnes+x14residence+x15worryaboutacademicperformance+x16sleepinghours+x17familyeconomiclevel+x18chronicdisease+x19monthlyallowance+x20maritalstatus+x21presenceofpartner+x22stessfullifeevent+x23socialsupport+x24PIU+X25insomnia+x26dpt+x27AADHD(y2)

Where x1*-x27* are coefficients for the corresponding variables. And the equation for **ADHD** was;

ADHD(Y2)=x1year+x2age+x3sex+x4cannabis+x5chat+x6currentalcoholuse+x7headtrauma+x8smoking+x9everalcoholuse+x10exercise+x12academicfailure+x13parentalpsychiatricillness+x18chronicdisease+x28mothereducation+x29fathereducation+x30birthorder+x31depression(y1)

Where *x1-13*, *x18*, and *x28-32*, are regression coefficients for the corresponding variables.

Checking for the rank condition is started by constructing a system matrix in which the endogenous variables of the structural model are listed on the left side of the matrix (rows) and all variables in the structural model (excluding disturbances) are listed along the top (columns). In each row, 0 or 1 appears in the columns that correspond to that row. “1” indicates that the variable has a direct effect on the endogenous variable. “1” also appears in the column that corresponds to the endogenous variable represented by that row. The remaining entries are “0”, which indicates the excluded variables. The rank condition must be evaluated for each equation of endogenous variables [81].

The steps to do so for a model with all possible disturbance correlations [81] were:

1. Begin with the first row of the system matrix (the first endogenous variable). Cross out all entries in that row and any column in the system matrix with 1 in this row. Prepare a reduced matrix from the remaining entries without row and column labels.
2. Simplify the reduced matrix further by deleting any row with entries that are all zeros and an exactly replicated row or that can be reproduced by adding other rows together. The number of remaining rows is the rank. Which is met for the equation of endogenous variables if the rank of the reduced matrix is greater than or equal to the total number of endogenous variables minus 1.
3. Repeat steps 1 and 2 for every endogenous variable. If the rank condition is satisfied for every endogenous variable, then the model is identified.

### Developing a system matrix

In our case, there were two endogenous variables. Therefore, 2 **minus** 1 = 1. In order to be identified, the above specified model should have a rank of 1 or more. In this case, there were only two rows, and there was no row with all entries of zero, the same row, or a row that can be reproduced by adding other rows. Therefore the complete system matrix seems;

$$\left[\begin{array}{ccccccccccccccccc} Outcome & x1 & x2 & x3 & x4 & x5 & x6 & x7 & x8 & x9 & x10 & x11 & x12 & x13 & x14 & x15 & x16\\ Y1 & 1 & 1 & 1 & 1 & 1 & 1 & 1 & 1 & 1 & 1 & 1 & 1 & 1 & 1 & 1 & 1\\ Y2 & 1 & 1 & 1 & 1 & 1 & 1 & 1 & 1 & 1 & 1 & 0 & 1 & 1 & 1 & 0 & 0 \end{array}\right]$$

$$\left[\begin{array}{ccccccccccccccc} x17 & x18 & x19 & x20 & x21 & x22 & x23 & x24 & x25 & x26 & x27 & x28 & x29 & x30 & x31\\ 1 & 1 & 1 & 1 & 1 & 1 & 1 & 1 & 1 & 1 & 1 & 0 & 0 & 0 & 0\\ 0 & 1 & 0 & 0 & 0 & 0 & 0 & 0 & 0 & 0 & 0 & 1 & 1 & 1 & 1 \end{array}\right]$$

Then the next step is applying the above mentioned procedures for each equation separately.

Evaluation for depression (Y1)

$$\left[\begin{array}{ccccccccccccccccc} Factors & x1 & x2 & x3 & x4 & x5 & x6 & x7 & x8 & x9 & x10 & x11 & x12 & x13 & x14 & x15 & x16\\ Y1 & strike1 & strike1 & strike1 & strike1 & strike1 & strike1 & strike1 & strike1 & strike1 & strike1 & strike1 & strike1 & strike1 & strike1 & strike1 & strike1\\ Y2 & strike1 & strike1 & strike1 & strike1 & strike1 & strike1 & strike1 & strike1 & strike1 & strike1 & strike0 & strike1 & strike1 & strike1 & strike0 & strike0 \end{array}\right]$$

$$\left[\begin{array}{ccccccccccccccc} x17 & x18 & x19 & x20 & x21 & x22 & x23 & x24 & x25 & x26 & x27 & x28 & x29 & x30 & x31\\ strike1 & strike1 & strike1 & strike1 & strike1 & strike1 & strike1 & strike1 & strike1 & strike1 & strike1 & strike0 & strike0 & strike0 & strike0\\ strike0 & strike1 & strike0 & strike0 & strike0 & strike0 & strike0 & strike0 & strike0 & strike0 & strike0 & 1 & 1 & 1 & 1 \end{array}\right]$$

Rank of the reduced matrix $\left[\begin{array}{ccccc} 1 & 1 & 1 & 1 & 1 \end{array}\right]$ = **1**, so depression equation is **identified.**

Evaluation for ADHD (Y2)

$$\left[\begin{array}{ccccccccccccccccc} Factors & x1 & x2 & x3 & x4 & x5 & x6 & x7 & x8 & x9 & x10 & x11 & x12 & x13 & x14 & x15 & x16\\ Y1 & strike1 & strike1 & strike1 & strike1 & strike1 & strike1 & strike1 & strike1 & strike1 & strike1 & strike1 & strike1 & strike1 & strike1 & strike1 & strike1\\ Y2 & strike1 & strike1 & strike1 & strike1 & strike1 & strike1 & strike1 & strike1 & strike1 & strike1 & strike0 & strike1 & strike1 & strike1 & strike0 & strike0 \end{array}\right]$$

$$\left[\begin{array}{ccccccccccccccc} x17 & x18 & x19 & x20 & x21 & x22 & x23 & x24 & x25 & x26 & x27 & x28 & x29 & x30 & x31\\ 1 & strike1 & 1 & 1 & 1 & 1 & 1 & 1 & 1 & 1 & 1 & strike0 & strike0 & strike0 & strike0\\ strike0 & strike1 & strike0 & strike0 & strike0 & strike0 & strike0 & strike0 & strike0 & strike0 & strike0 & strike1 & strike1 & strike1 & strike1 \end{array}\right]$$

Rank of the reduced matrix $\left[\begin{array}{ccccccccccccc} 1 & 1 & 1 & 1 & 1 & 1 & 1 & 1 & 1 & 1 & 1 & 1 & 1 \end{array}\right]$ = **1** so, the ADHD equation is **identified.** Since the rank of the equation of every endogenous variable of each system matrix equals the number of endogenous variables minus 1 (i.e., 2 minus 1), the rank condition is satisfied. Thus, the non-recursive model specified in **Fig 2** is identified.

### Instrument or instrumental variable

It is a variable that has a direct effect on the problematic causal variable but no direct effect on the outcome variable [81]. The instrument variable approach can also be used to solve omitted variable bias and other kinds of endogeneity problems [78]. In our non-recursive SEM, the disturbance variables for depression and ADHD were correlated; therefore, we need instruments for both variables. Thus, worry about academic performance, a history of stressful life events, social support, problematic internet use, and insomnia were used as instrumental variables for the depression equation. While mother education level, father education level, and birth order were used as instrumental variables for ADHD.

**3. Model estimation**

It is a process of obtaining numerical values for unknown (free) parameters [82]. For this study, the estimation method was maximum likelihood with 3500 bootstrap samples since the data didn’t meet the multivariate normality test.

**4. Evaluation of model fit (model testing)**

In SEM, each path coefficient’s evaluation is based on the fit indices. Indices such as the Chi-square test (χ2) [83], the goodness-of-fit index (GFI) [80,83], and the normed fit index (NFI) [84] are highly sensitive to sample size. Others, like the Root mean square error of approximation (RMSEA), comparative fit index (CFI) [80,83,85], and Tucker-Lewis index (TLI) [86], are less sensitive to sample size. Information criteria (AIC and BIC) are not useful in testing null hypotheses but are useful for selecting models with the least overfitting. BIC is also an estimation of how parsimonious a model is among candidate models [84]. In our case, model fit indices that are sensitive to sample size were not used. Thus, RMSEA, CFI, TLI, AIC, and BIC were used for model selection. Though none of them serve as a golden rule, there are recommended values [84] for each fit index. Thus, RMSEA < 0.05 [85], CFI, and TLI (NNFI) > 0.90 [80,83,85] indicate better model fitness.

**5. Model modification**

It involves adjusting a specified and estimated model by either freeing parameters that were fixed or fixing parameters that were free. The modification index (MI) and the standardized expected parameter change (SEPC) were used to aid in the selection of parameters to be added to a model in order to improve the model fit. If fixed parameters associated with a large MI value > 4 with 1 degree of freedom at an alpha level of 0.05 were examined to decide whether they were theoretically plausible to include in the model and be freely estimated.

### Ethical consideration

Ethical clearance was obtained from the institutional review board of UoG under reference number Ref No. /IPH/2131/2014. Permission was obtained from the UoG main registrar’s office in order to get a list of students’ identification numbers. A Permission letter from each college, institute, faculty, school, or department was obtained in order to collect the data from students at any time. A written informed consent was obtained from each participant prior to the administration of questionnaires. Any personal identifiers were not recorded during the data collection, and other recorded information was kept in a way that assures data anonymity and confidentiality. And also, confidentiality was maintained during all phases of research activities. Participants were informed that their participation in the study was completely voluntary, and they also had the right to withdraw themselves at any time.

## Results

### Socio demographic factors

The total number of distributed questionnaires was 1544, and of those, 1514 were completed, yielding a response rate of 98.1%. Among the participants, about two-thirds (65%) were male. The median age of the participants was 21 years (IQR = 2). Nearly all (96.9%) of the respondents were unmarried. Approximately two-thirds (66.8%) had previously lived in urban areas. Nearly Four-fifths (79.7%) reported that they have a closely related stable partner (**Table 1**).

**Table 1:** Socio-demographic characteristics of respondents, UoG, Northwest Ethiopia, 2022.

Variable	Category	Frequency (n = 1514)	Percentage (%)
Age	[Median = 21 ± IQR(2) years		
Sex	Male	986	65.1
	Female	528	34.9
Marital status	Single	1467	96.9
	Married	40	2.6
	Divorced	4	0.3
	Lives separately	3	0.2
Residence	Rural	502	33.2
	Urban	1,012	66.8
Mother’s education	Unable to read and write	463	30.6
	Primary	413	27.3
	Secondary	258	17.0
	Higher (college and above)	380	25.1
Father’s education	Unable to read and write	239	15.8
	Primary	476	31.4
	Secondary	242	16.0
	Higher (college and above)	557	36.8
Monthly allowance (n = 1504)	**[**Median = 1000 ± IQR(1000) ETB		
Family economic level	Low	277	18.3
	Medium	1191	78.7
	Rich	46	3.0
Presence of stable partner	No	308	20.3
	Yes	1206	79.7

#### Health and behavioral related factors

More than half (58.5%) of the study participants reported that they had experienced stressful events within one month prior to data collection. Most of the participants (91.7%) and 92.3% reported that they had no history of parental psychiatric problems or chronic diseases, respectively. Most of the participants (93.4%) and 89.8% explained that they did not experience smoking cigarettes or chewing Khat, respectively (**Table 2**).

**Table 2:** Clinical and behavioral characteristics of respondents, UoG, Northwest Ethiopia, 2022.

Variable	Category	Frequency (n = 1514).	Percentage (%)
Birth order	Median = 2 IQR = 3		
Presence chronic disease	No	1398	92.3
	Yes	116	7.7
Parental history of psychiatric illness	No	1389	91.7
	Yes	125	8.3
History of head trauma	No	1369	90.4
	Yes	145	9.6
History of stressful life events	No	885	58.5
	Yes	629	41.6
History of smoking	No	1414	93.4
	Yes	100	6.6
History of alcohol use	No	507	33.5
	Yes	1,007	66.5
Current alcohol use	No	769	50.8
	Yes	745	49.2
History of chat chewing	No	1360	89.8
	Yes	154	10.2
History of cannabis use	No	1467	96.9
	Yes	47	3.1
Physical exercise	No	388	25.6
	Yes	1126	74.4
PIU	Mean = 23.2 ± (SD = 8.4)		
Insomnia	Median = 7 ± (IQR = 7)		
Social support	Mean = 10.3 ± (SD = 2.2)		

### Education related factors

In terms of academic-related factors, more than half (58.7%) of the study participants reported that they worried about their academic performance. The median sleeping and studying hours were also reported as 8 and 5, respectively (**Table 3**).

**Table 3:** Academic related characteristics of respondents, UoG, Northwest Ethiopia, 2022.

Variable	Category	Frequency(n = 1514)	Percentage (%)
Year of study	I	447	29.5
	II	292	19.3
	III	465	30.7
	IV	207	13.7
	V	46	3.0
	VI	57	3.7
History of academic failure	No	1412	93.3
	Yes	102	6.7
Worrying about academic performance	No	629	41.5
	Yes	885	58.5
Average number of sleeping hours/day	Median = 8 hrs., IQR = 3		
Average number of reading hours/day	Median = 5 hrs., IQR = 2		

**Fig 3:**
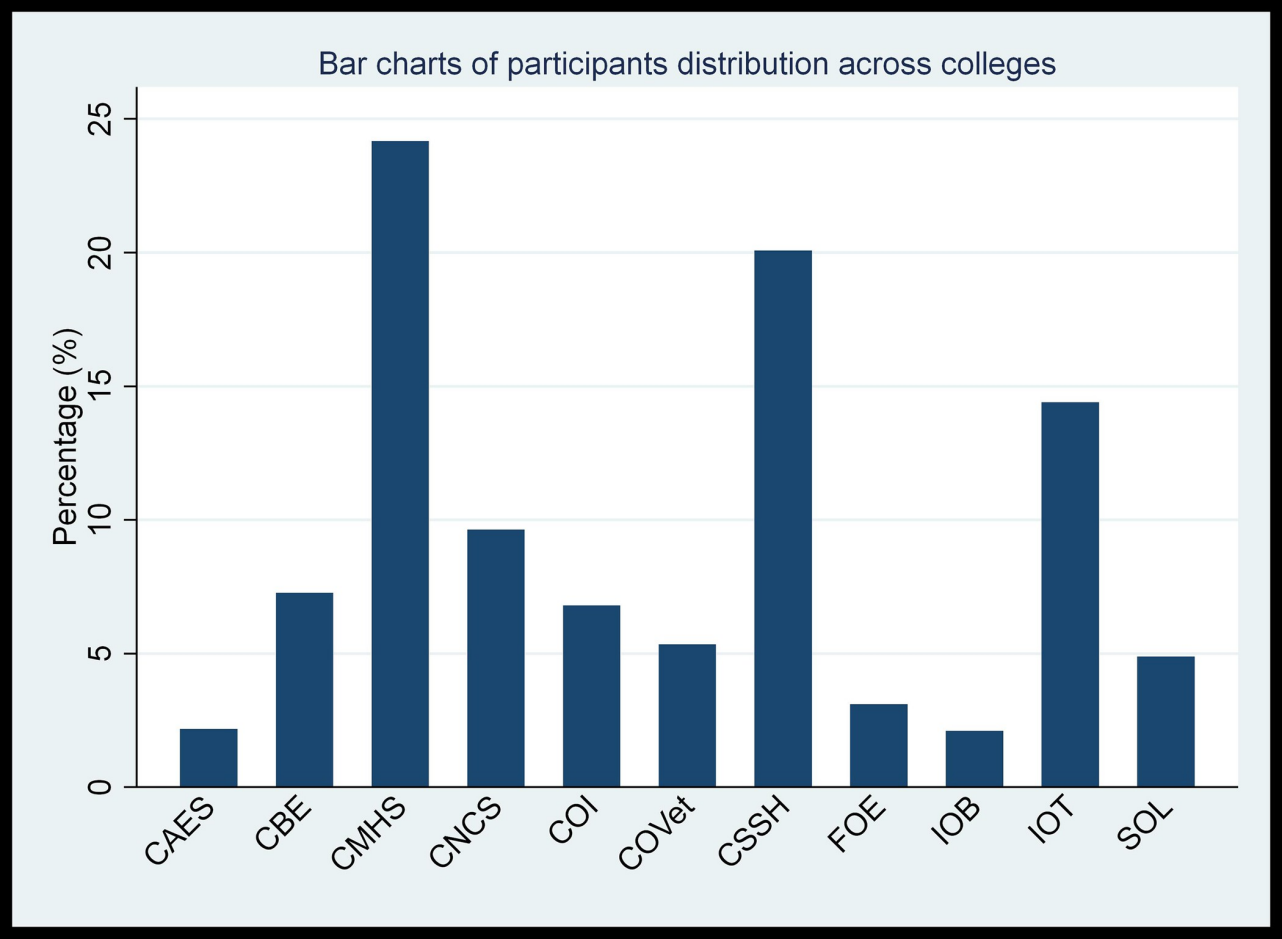
Bar chart of student’s distribution based on their college/institute/faculty/school, University of Gondar, Northwest Ethiopia, 2022. CAES- College of Agriculture and Environmental Science; CBE-College of Business and Economics; CMHS- College of medicine and health science; CNCS- College of natural and computational science; COI- College of Informatics; COVet- College of Veterinary medicine; CSSH- College of Social Science and Humanities; FOE- Faculty Of Education; IOB- institute of biotechnology; IOT-institute of technology and SOL-School Of Law.

As presented in **Fig 3,** approximately one-fourth of the respondents were from the College of Medicine and Health Science, and nearly one-fifths were from the College of Social Science and Humanities.

### Magnitude of depressive symptoms

From the total number of students who participated in the study, more than one-third (37.6%; 95% CI: 35.2–40.1%) had symptoms consistent with depression. In terms of severity, 36.3% had mild, 22.5% had moderate, 10.6%, and 4.6% had moderately severe and severe depressive symptoms, respectively. Of the total participants, 482 (31.8%), 496 (32.8%), 677 (44.7%), and 810 (53.5%) reported that they never had a feeling of little interest in doing things, being tired, having a poor appetite or overeating, and having a problem of moving or speaking, respectively. And nearly three-fourths (73.7%) reported that they had never thought about hurting themselves. However, 495 (32.7%) had the problem of concentrating on things for several days (**S3 Table)**.

### Magnitude of ADHD symptoms

From the total respondents, 20.01% were at high risk of ADHD (303/1514; 95% CI: 18.1–22.1%). A total of 490 participants (32.4%) reported that sometimes they had difficulty wrapping up the final details of their project or assignment after completing challenging parts; 525 (34.7%) and 518 (34.4%) reported that they often had difficulty organizing things and problems remembering appointments or obligations, respectively. And 529 (34.9%), 470 (31.0%), and 593 (39.2%) sometimes avoid or delay tasks when they require a lot of thought; fidget when they sit for a long time; and feel overly active, respectively **(S4 Table)**.

### Assumptions for structural equation modeling

#### Sample size adequacy and sphericity

The Kaiser-Meyer-Olkin (KMO) measure of sampling adequacy and Bartlett’s test of sphericity were checked. The overall KMO measure of sampling adequacy result was 0.90, and Bartlett’s test of sphericity for all constructs was significant (P < 0.001), which indicates a good partial correlation exhibited in the data (**Table 4**).

**Table 4:** The KMO and Bartlett’s test of sphericity results, UoG, Northwest Ethiopia, 2022.

Constructs	KMO	Bartlett’s Test of Sphericity	
		Approx. Chi-Square	P—value
ADHD	0.79	2189.7	0.00
Depression	0.89	3286.2	0.00
Social support	0.66	698.8	0.00
Insomnia	0.81	2647.8	0.00
PIU	0.91	7065.4	0.00
Overall	0.90	17231.5	0.00

#### Multivariate normality, missing and outliers

In SEM, one of the main concerns is whether the data has a multivariate normal distribution, because that determines the method of estimation and to what extent the estimates obtained from the most common methods are trustworthy. A multivariate normal distribution (MVND) considers that each individual variable in a sample has a univariate and joint (bivariate) normal distribution [87]. This assumption is very critical, especially for estimation in SEM with ML [81]. If the data does not have MVND and ML estimation is used, it can cause underestimation of standard errors of parameter estimates, leading to inflated statistics and hence possibly erroneous attributions of the significance of specific relationships in the model [88]. In our case, it is also evidenced that the MVND, which was assessed with the help of Mardia’s skewness and kurtosis test, the Henze-Zirkler test, and the Doornik-Hansen test, was not satisfied.

Outliers are those data points that deviate from the global behavior of the majority of data. It can be due to measurement or recording errors; some of them can represent something significant from the viewpoint of the application domain. Thus, removing it may result in loss of useful information and lead to biased results. A good outlier might be an atypical but legitimate pattern, while a bad outlier might be a “garbage” pattern. Using robust analysis methods for this type of data is more acceptable than removing all outliers, which can increase the error rate [89]. In our case, outliers were checked using Mahalanobis distance. Usually, if the probability of the Mahalanobis distance is less than 0.001 [90], then the observation is considered a multivariate outlier. On this, 77 observations indicate an outlier. Measurement errors and errors in data entry were checked. And finally cross-checked with the hard copy. However, all the measurements were logical, and we considered them good outliers.

Regarding missing values, there were ten observations with missing values. All missing values were from predictors and had no relationship with the outcome variable; thus, list-wise deletion was performed because they constituted less than 5% of the total sample and were considered missing completely at random.

#### Independence of observation

The intraclass correlation coefficient (ICC) is one mechanism for deciding whether to use a hierarchical model [91], which explains the variance of the outcome due to variability in clusters or groups. The large magnitude of ICC shows the need for a hierarchical model. Literature shows that an ICC of 0.1 and above is high, and thus we would need multilevel modeling to avoid Type 1 errors [92]. A Decrease in both the number of subjects per cluster and the number of clusters is associated with a decrease in statistical power for non-null variance. The number of clusters required is also dependent on whether the researchers are interested in interpreting the specific level two groups or doing random effect models that need generalizability to the population represented by the clusters [91]. To learn more about the statistical problems, clustering at the college and department level was checked, and the ICC level was much lower than the required cut-off point (0.1) **(S5 Table).**

#### Common method bias

Despite the fact that data collection using self-administered questionnaires has become popular in recent years, the majority of researchers do not make mention of common method bias (CMB) and thus do not report whether they have tested their construct for CMB or, at least, acknowledge it as a limitation to their research [93]. It is more closely related to the structure of the questionnaire and can occur when both the independent and dependent variables are measured using the same response method (e.g., ordinal scales) unless the study is experimental or based on a secondary data collection method, where CMB is less common [94].

In our study, outcome variables (ADHD and depression) and other exogenous latent variables (social support, insomnia, and problematic internet use) were measured using a common method (i.e., an ordinal scale). It can be evidenced that CMB is present if exploratory factor analysis, with all primary study variables included, results in one factor accounting for more than 50% of the variance or if CFA suggests that the one-factor model fits the data as well as the proposed model [94]. Therefore, Harman’s single factor test for CMB was conducted and declared that there is no CMB problem in our study since the variance explained by a single factor was 19%.

#### Instrumental variables validity and strength

Instrumental variables are one method of ensuring the identification status of an equation in non-recursive SEM. Identification test (Anderson canon. corr. LM statistic): Weak identification test (Cragg-Donald Wald F statistic), strength of instrumental variables (Stock-Yogo weak ID test critical values), and validity of instrumental variables (Sargan statistics) were checked. The Cragg-Donald Wald F statistic shows the maximum F statistic at each maximal instrumental variable bias. If F-statistic is greater than all maximal IV relative bias for Stock-Yogo weak identification test critical values, then we can conclude that instrumental variables in the model were strong. In our case, the maximal IV relative bias for the Stock-Yogo weak identification test critical values for ADHD was 22.30. However, the corresponding F-statistic was 30.8, which is greater than 22.3. Therefore, the instrumental variables for ADHD were strong. And the maximal IV relative bias for Stock-Yogo weak identification test critical values for depression was 26.9. However, the corresponding F-statistic was 102.6. Therefore, instrumental variables for depression were also strong (**S6 Table).**

#### Stability of the non-recursive system

Stability analysis of simultaneous equation systems revealed an eigenvalue stability index of 0.26, which showed that all eigenvalues lie inside the unit circle. Therefore, we can conclude that SEM meets the stability condition.

#### Confirmatory factor analysis

**Fig 4:**
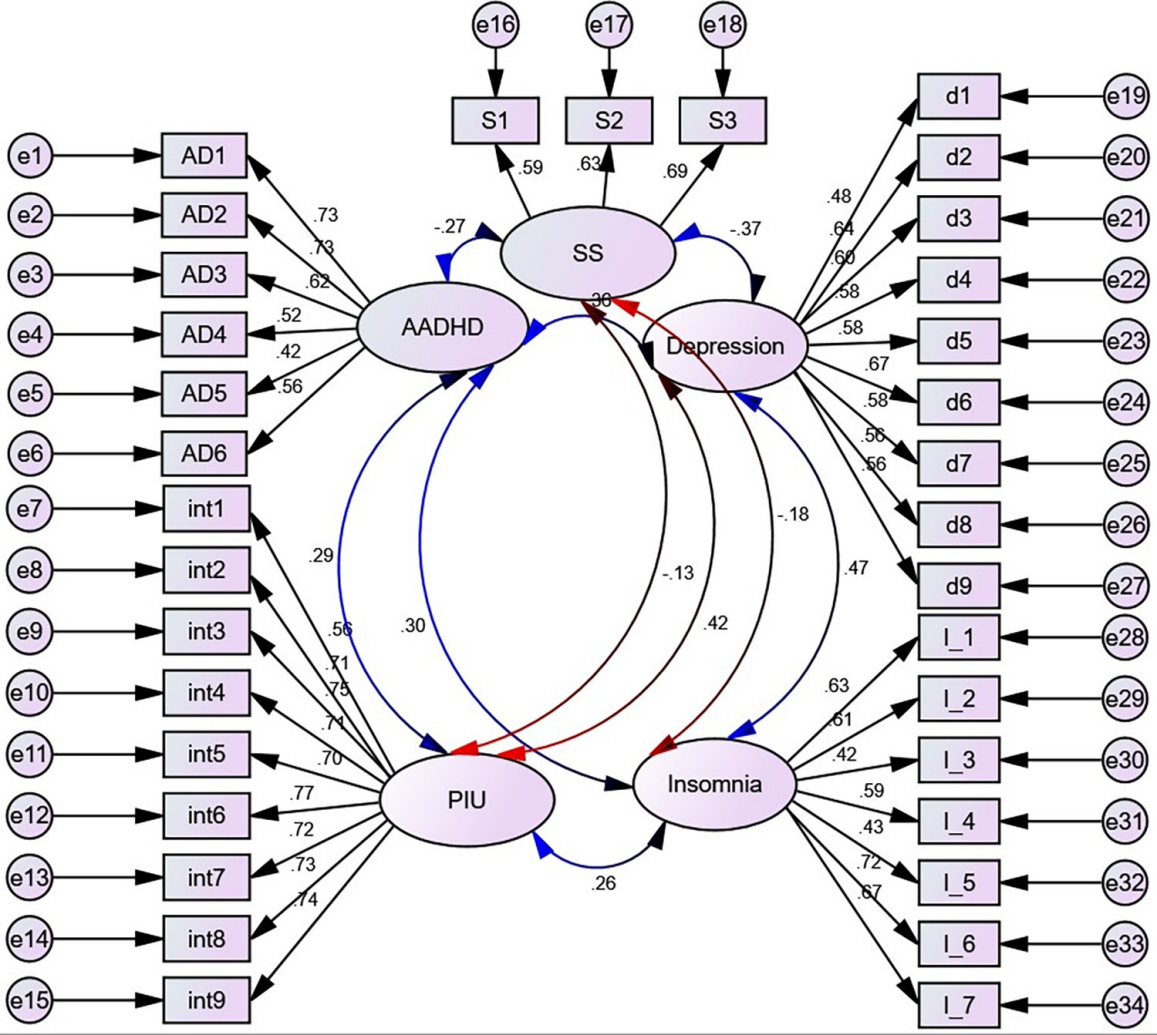
Initial measurement model with standardized estimates displayed, UoG, Northwest Ethiopia, 2022. Circles indicate latent variables or error terms, rectangles indicate observed variables, single arrows indicate factor loadings or regression coefficients, and double arrows indicate the covariance between latent variables.

Measurement models for hypothesized and empirically verified factor structures in our SEM were first evaluated through confirmatory factor analysis (CFA) (**Fig 4**). Indicators have been allowed to load on factors, and residuals and errors have been allowed to correlate (if these indicators are believed to have common causes other than the latent factors included in the model). Then the structural relations of the latent factors are modeled by the combination of CFA models with structural path models on the latent constructs, which represents our general SEM framework.

Model fit indices (TLI = 0.87, CFI = 0.88) for the measurement model were below the required cutoff point of 0.9 even if other goodness of fit measures (CMIN/DF = 4.99, RMSEA (P-value) = 0.05 (0.10) were at the required level. Besides, AIC and BIC were 2738.24 and 2741.96, respectively. This indicates that the CFA of the initial models is poor and that the analysis is not supported by the sample variance-covariance data. Therefore, we continued to add possible modifications based on MI and SEPC. Some covariances between error terms within construct indicators were added (**S1 Fig).** After that, all the model fit measures become acceptable (CMIN/DF = 2.97, TLI = 0.93, CFI = 0.94, RMSEA (P-value) = 0.04 (1.00)), AIC = 1678.48, and BIC = 1682.73). However, the model was not parsimonious. Therefore, we applied average partial factorial parceling.

### Item parceling

Average partial factorial parceling was applied to three constructs such as ADHD, depression, and insomnia. Mostly, the recommended number of parcels is 3 [95]. Thus, each construct had three parcels **(S8 Table).** Regarding model fitness, the majority of model fit indices were at the required level (CMIN/DF = 7.24, TLI = 0.91, CFI = 0.92, RMSEA (P-value) = 0.06 (0.00), AIC = 1400.3, and BIC = 1676.75). However, we continued to add possible modifications based on MI and SEPC to improve model fit indices (**S2 Fig)**.

Finally, all goodness of fit indices were in an acceptable range (CMIN/DF = 3.47, TLI = 0.96, CFI = 0.97, RMSEA (P-value) = 0.04 (1.00), AIC = 711). Model parsimony, which can be expressed by BIC, was also significantly reduced from 1676.8 to 713.8. Thus, we decided to proceed with the modified measurement model with parceled items.

### Structural model

**Fig 5:**
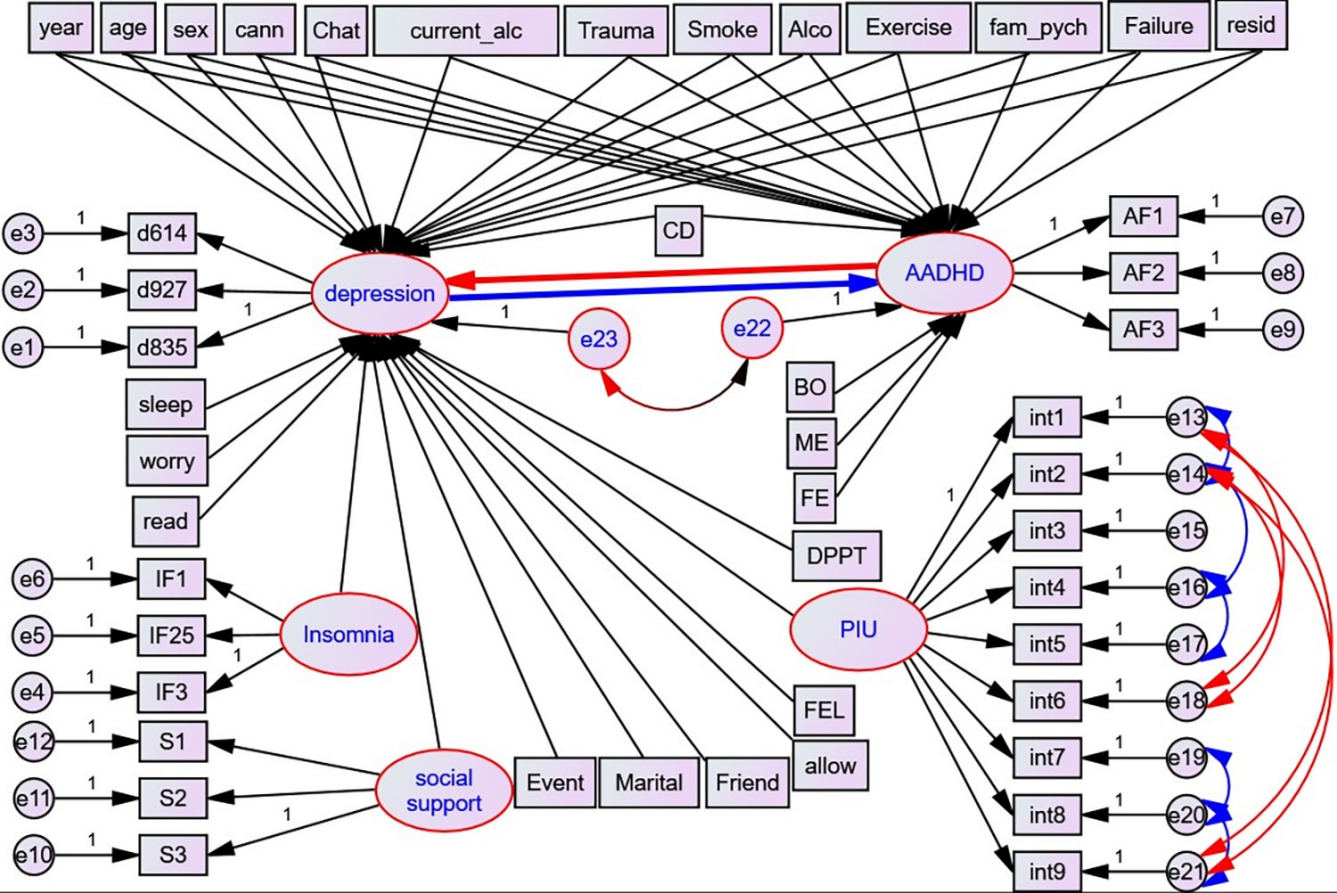
Full structural model after parceling, UoG, Northwest Ethiopia, 2022. AF1-3- represents parcel 1–3 for ADHD, IF1, 1F25, IF34- represents parcel 1–3 for insomnia and d614, d927 and d835 represents parcel 1–3 for depression respectively; exercise = Physical exercise, year = year of study, cann = cannabis, current_alco = current alcohol, alco = alcohol use, failure = history of academic failure, worry = worry about academic performance. Event = history of stressful life event, read and sleep = reading and sleeping hours per day, ME = mother education level, BO = birth order, PIU = Problematic Internet Use, int1-9 = items for Problematic Internet Use, DPTT = department type and CD = Chronic Disease.

A structural model is a statistical technique that allows a set of relationships between one or more independent variables and outcome variables to be examined. Both dependent variables and independent variables can be either factors or measured variables. In our case, the structural model was non-recursive (**Fig 5)**.

#### Model selection

After full SEM, a model that represented the data relatively well was selected. First, all insignificant variables were removed from the model and compared to the full model, then error terms of the indicators within the same construct were included, and finally, correlation between independent predictors was allowed. A comparison was made using fit indices and information criteria **(Table 5)**.

**Table 5:** Model selection from possible candidate models after parceling, UoG, Northwest Ethiopia, 2022.

Model	AIC	BIC	CFI	TLI	RMSEA (P-value)
Hypothesized model	8261.6	8856.1	0.65	0.63	0.07(0.00)
Model with significant predictors only	4289.1	4687.2	0.79	0.77	0.07(0.00)
Model with significant predictors and covariance between indicators error terms.	3501.5	3968.5	0.83	0.82	0.06(0.00)
Model with significant predictors and covariance between indicator error terms and between exogenous variables.	1667.6	2102.8	.93	.92	0.04(1.00) **Selected**

### Factors associated with depressive symptoms

**Fig 6:**
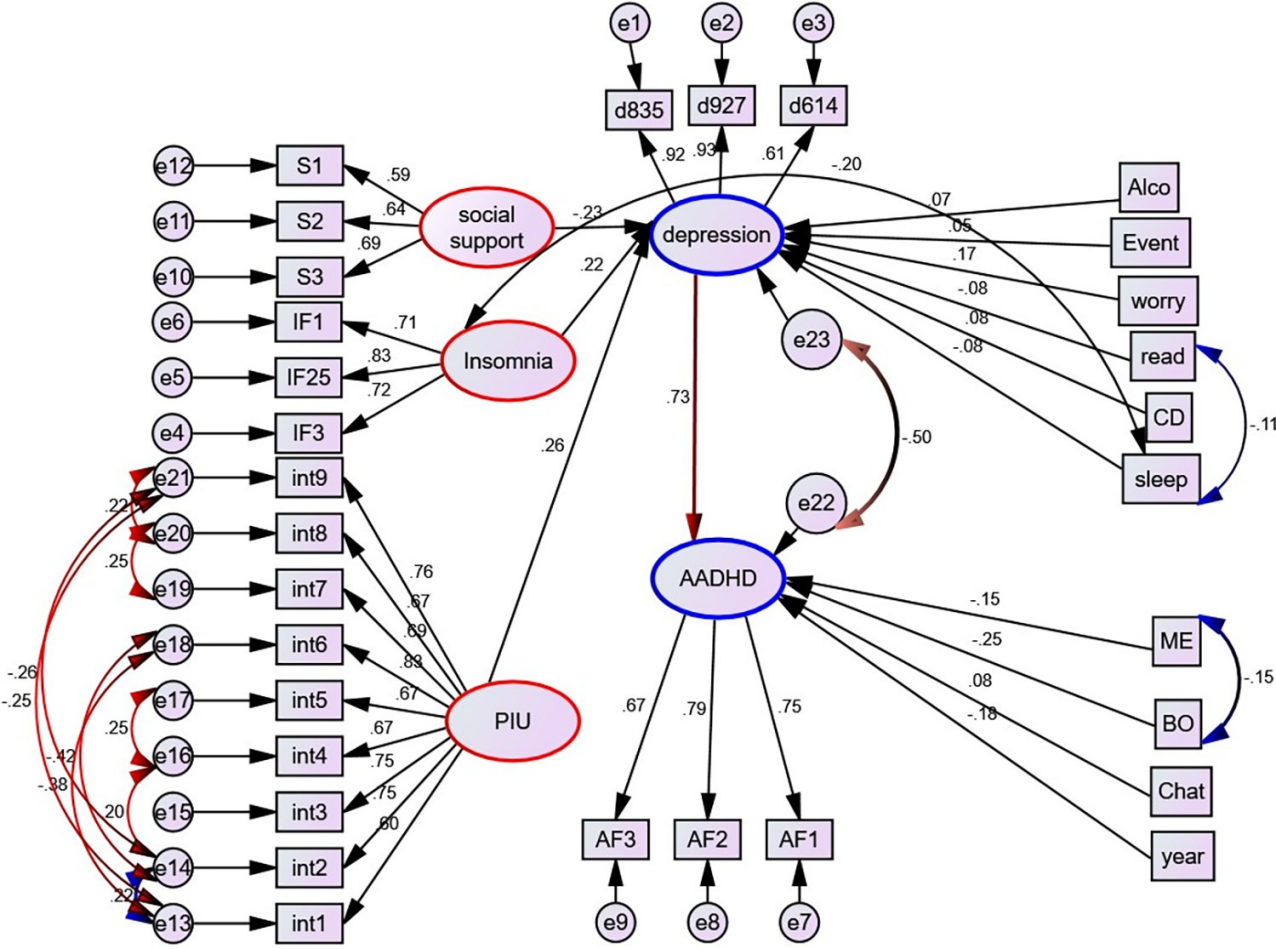
Final SEM showing factors associated with Attention Deficit Hyperactivity Disorder and depressive symptoms, UOG, Northwest Ethiopia, 2022. Standardized estimates are displayed.

Following the development of SEM, variables such as age, sex, cannabis use, current alcohol use, residence, marital status, presence of partners, history of parental psychiatric problems, father education level, average monthly allowance, history of smoking, physical exercise, department type, history of academic failure, and self-reported family economic level were removed from the model. Since there was no statistically significant direct and/or indirect effect on either outcome variable. Then the model is summarized after all remaining variables have been controlled and the path coefficients have been adjusted. In addition, the feedback loop that runs from ADHD to depressive symptoms was also removed since it was statistically insignificant (**Fig 6)**.

Our SEM revealed that the presence of medically diagnosed chronic disease, history of head injury, history of stressful life events, alcohol use, worrying about academic performance, number of sleeping hours, number of studying hours, social support, PIU, and insomnia were significantly associated with the depressive symptoms score. Thus, after controlling for other variables in the model, the presence of a diagnosed chronic illness had a significant direct effect [β = 0.19, 95% CI: 0.09, 0.30] on depressive symptoms. Having stressful life events had a positive direct effect [β = 0.07, 95% CI: 0.01, 0.13] on depressive symptoms after controlling other variables in the model in comparison to those without these events. Alcohol use also had a significant positive direct effect [β = 0.10, 95% CI: 0.03, 0.17] on depressive symptoms compared to non-users. Being worried about academic performance had a direct positive effect [β = 0.24, 95% CI: 0.18, 0.30] on depressive symptoms.

The model likewise demonstrated that social support [β = -0.23, 95% CI; -0.29, -0.17], average number of sleeping hours [β = -0.03, 95% CI; -0.04, -0.01], and average number of studying hours per day [β = -0.02, 95% CI; -0.04, -0.01] had a direct negative effect on depressive symptoms, controlling other variables in the model. While PIU [β = 0.23, 95% CI; 0.18, 0.27] and insomnia [β = 0.24, 95% CI; 0.17, 0.30] had a positive direct effect on depressive symptoms **(Table 6).**

**Table 6:** Factors associated with depressive symptoms, UoG, northwest Ethiopia, using SEM, 2022. Unstandardized estimates.

Variable	Adjusted estimates [95% CI]	SE
Medically confirmed chronic disease	0.19 [0.09, 0.30]**	0.06
Worry about academic performance	0.24 [0.18, 0.30]**	0.03
History of stressful life event	0.07 [0.01, 0.13]*	0.03
Ever alcohol use	0.10 [0.03, 0.17]*	0.03
Number of studying hours	-0.02 [-0.04, -0.01]**	0.01
Number of sleeping hours	-0.03[-0.04, -0.01]*	0.01
PIU	0.23 [0.18, 0.27]**	0.02
Social support	-0.23 [-0.29, -0.17]**	0.03
Insomnia	0.24 [0.17, 0.30]**	0.03

CI- Confidence Interval and SE- Standard Error.

^**a**^ “*”- indicates statistically significant at P- value <0.05, “**”-indicates statistically significant at P- value < 0.001.

### Factors associated with ADHD

Year of study, mother education level, birth order, chat chewing, and depressive symptoms had a statistically significant direct effect on ADHD symptoms, while the presence of medically confirmed chronic disease, worrying about academic performance, history of stressful life events, history of academic failure, alcohol use, history of previous head injury, average number of sleeping and studying hours, social support, PIU, and insomnia had a depression-mediated indirect effect on ADHD.

Thus, the year of study had a negative direct effect [β = -0.10, 95% CI: -0.12, -0.03] on ADHD. And also, mother education level [β = -0.09, 95% CI: -0.13, -0.06] and birth order [β = -0.09, 95% CI: -0.11, -0.07] had a significant negative direct effect on ADHD. Chat chewing and depression scores had a direct positive influence on ADHD, with adjusted estimates of [β = 0.18, 95% CI: 0.06, 0.30] and [β = 0.73, 95% CI: 0.62, 0.86], respectively.

Regarding indirect effects, the presence of medically confirmed illness [β = 0.14, 95% CI: 0.06, 0.22], being worried about educational performance [β = 0.18, 95% CI: 0.13, 0.23], history of stressful life events [β = 0.05, 95% CI: 0.003, 0.10], ever alcohol use [β = 0.07, 95% CI: 0.03, 0.12], PIU [β = 0.16, 95% CI: 0.13, 0.21], and insomnia [β = 0.17, 95% CI: 0.13, 0.23] implied a statistically significant indirect positive effect on ADHD. While social support [β = -0.16, 95% CI: -0.22, -0.12], average number of sleeping hours [β = -0.02, 95% CI: -0.03, -0.01], and average number of studying hours [β = -0.02, 95% CI: -0.03, -0.01] had a negative indirect effect on ADHD (**Table 7**).

**Table 7:** The direct and indirect effects of socio-demographic, clinical and behavioral factors on ADHD, UoG, Northwest Ethiopia, using SEM, 2022. Unstandardized estimates.

Variable	Adjusted Direct effect (95% CI)	SE	Adjusted Indirect effect (95% CI)	SE
Year of study	-0.10[-0.12, -0.03]*	0.01	----	----
Mother education level	-0.09[-0.13,-0.06] *	0.02	----	----
Birth order	-0.09 [-0.11, -0.07]*	0.01	----	----
Chat chewing	0.18[0.06, 0.30]**	0.06	----	----
Depressive symptoms	0.73[0.62, 0.86]	0.06	----	----
Presence of chronic disease	----	----	0.14 [0.06, 0.22]**	0.04
Worrying about academic performance	----	----	0.18 [0.13, 0.23]**	0.03
History of stressful life event	----	----	0.05[0.003, 0.10]*	0.02
Ever alcohol use	----	----	0.07 [0.03, 0.12]*	0.02
Number of sleeping hours	----	----	-0.02 [-0.03, -0.005]*	0.01
Number of studying hours	----	----	-0.02 [-0.03, -0.01]**	0.01
Social support	----	----	-0.16 [-0.22, -0.12]**	0.03
PIU	----	----	0.16 [0.13, 0.21]**	0.02
Insomnia	----	----	0.17 [0.13, 0.23]**	0.03

CI- Confidence Interval and SE- Standard Error.

^**a**^ “*”- indicates statistically significant at P- value <0.05, “**”- indicates statistically significant at P- value < 0.001.

In addition, the covariances between insomnia and sleep hours (covariance = -0.27, 95% CI: -0.43 to -0.15), mother education level and birth order (covariance = -0.36, 95% CI: -0.48, -0.25), and sleep and studying hours (covariance = -0.77, 95% CI: -0.88, -0.66) were statistically significant and different from zero. This indicates that the variables are negatively correlated with each other.

## Discussion

This study examined the magnitude relationship and determinants of depression and ADHD among UoG undergraduate students, Northwest Ethiopia using non-recursive SEM (simultaneous estimation of the feedback loop between depression and ADHD symptoms). And we found that the prevalence of depression and ADHD symptoms was considerable and estimated to be 37.6% (95% CI: 35.2–40.1%) and 20.01% (95% CI: 18.1–22.1%) respectively. Independent factors such as medically confirmed chronic disease, history of head injury and stressful life events, alcohol use, worrying about academic performance, number of sleeping and studying hours, social support, PIU, and insomnia were directly and indirectly related to depression and ADHD symptoms, respectively. While year of study, mother’s education level, birth order, chat chewing, and depressive symptoms had a direct effect on ADHD symptoms.

### Magnitude and determinants of depressive symptoms

The overall prevalence of depressive symptoms was found to be 37.6%. This figure is higher than previous reports from Ethiopian Universities [48,49,52,96,97]. This variation might be due to variations in screening instruments and study populations. For instance, studies conducted at Wolkite [52] and Bench-Sheko Zone, southwest Ethiopia [49], used the Depression Anxiety and Stress Scale-21 (DASS-21), and a study conducted at UoG [96] used the Beck Depression Inventory (BDI-II) scale and was conducted among medical students. A study conducted at Dilla University [97] also used the Hamilton Depression Rating Scale (HDRS), which assesses depressive symptoms over the past week. In addition, a study conducted in the Bench-Sheko Zone was community-based. Thus, economic stress, distance from the family, and problems of dealing with roommates combined with the presence of high social support from family compared to those living in the University may lower the prevalence of depression [55].

However, our finding is lower than previous findings from Arsi University [55] and the Amhara region [50], where the prevalence was reported as 52.3% and 77.2%, respectively. This might be due to the fact that the former was conducted among medical students only and the latter one was conducted during the early phase of the COVID-19 lockdown, which may increase the prevalence. Moreover, the tool used in the two studies was DASS-21, which can assess depression symptoms over the past week. Our study also found a higher magnitude of depressive symptoms compared to studies at universities outside Ethiopia, like in Uganda, Bangladesh, Malaysia, and Spain [51,53,56,57]. This might be due to the reason explained, or there may be a true difference in the prevalence of depression. However, our finding is in line with other studies conducted in Cape Town and China [98,99].

### Factors associated with depressive symptoms

The presence of a medically confirmed chronic illness had a direct positive effect [β = 0.19, 95% CI: 0.09, 0.30] on depressive symptoms. Which indicates students with medically confirmed chronic illness had higher levels of depression symptom scores as compared to their counterparts, keeping other variables in the model controlled. Similar findings were reported from a study conducted in Ethiopia [49] and other studies outside of Ethiopia, like in Turkey [100]. This might be due to chronic illness, which requires lifestyle adjustments with the treatment schedule that may affect the students’ freedom of mobility and independence. That strict lifestyle may be stressful and can cause despair or sadness [101].

Alcohol use had a significant positive direct effect [β = 0.10, 95% CI: 0.02, 0.15] on depression symptoms when other variables in the model were controlled. This indicates that having a history of alcohol use increases the level of depression symptoms by 0.10 compared to their counterparts. This finding is comparable with a study conducted at Debre Birhan University [48]. This can be explained as alcohol consumption can affect the brain and endocrine system (serotonin and other neurotransmitters in the brain) and predispose students to mental illness. In addition, alcohol increases stress hormones by activating the hypothalamus-pituitary-adrenal axis to produce excess cortisol [102]. However, another study conducted among UoG students revealed that alcohol and depression symptoms were not related [103]. The reason might be that the previous study only considered alcohol users during the study period (current users only), while the current studies assessed both the previous history and the current users, so our finding is ever-use of alcohol. A discrepancy in sample size and population may also contribute, where the previous study used 383 samples from medical and health science departments only.

Worry about academic performance had a direct positive effect [β = 0.24, 95% CI: 0.18, 0.30] on depressive symptoms. Being worried about academic performance increases depressive symptoms by 0.24. Which is consistent with studies conducted at UoG [52] and Makerere University, Uganda [53]. This might be explained by the relationship between working memory and education. Depression affects the working memory, which negatively affects the student’s test performance, which may make students worry about their academic performance [104]. And depression may affect students’ reading abilities, which in turn prevent them from achieving the responsibilities given to them by their parents.

Social support has a direct negative effect [β = -0.23, 95% CI: -0.29, -0.17] on depressive symptoms. When social support scores increase, scores for depressive symptoms decrease. This might be due to the disease nature of depression, since it has a feeling of inwardness and isolation. It is a huge energy-draining disorder. Thus, people with depression may fail to reach out and connect with others. Therefore, isolating themselves may deteriorate their symptoms. However, it has a positive role in mental health by helping individuals feel appreciated and connected with social networks [105]. A similar finding was reported by a study conducted at the bench Sheko zone and among Mizan Tepi University students from Ethiopia [49,106] and Iraq [58]. However, studies conducted at Jimma University and three northwest Ethiopian Universities, namely Debre Markos, Bahir Dar, and Debre Tabor, declared that social support had no relation to depressive symptoms [107,108]. This might be due to a difference in sample size (where the sample size of the previous study [107] was nearly one third of the current one), and the previous study fitted categorized data on social support while the current uses the continuous data as it is. Thus, dividing continuous variables into categories typically translates into losing power and correlation between variables, which can affect the effect size estimation [109]. The other study [108] was also conducted among first-year technology students and applied non-random sampling techniques. Thus, it cannot be generalized for the other populations included in our study.

The average number of sleeping hours per day had a direct negative effect [β = -0.03, 95% CI: -0.04, -0.01] on depressive symptoms. This indicates that when the number of sleeping hours increases, the level of depression decreases, and students who have fewer sleep hours are more likely to experience depressive symptoms. This finding is similar to a study conducted in Iraq [58]. Which can be explained as follows: when students get adequate sleep time, they may gain energy, keep their body healthy, and their ability to learn and remember may also increase. Moreover, poor sleep may create difficulties in regulating emotional well-being, which may leave them more vulnerable to depressive symptoms [110]. However, other studies conducted in Bangladesh [56] and Japan [111] reported that there was no association between depression symptoms and sleep duration. This might be due to the fact that the previous studies were conducted among freshman students, and the latter used the Japanese version of the Center for Epidemiologic Studies Depression Scale (CES-D) [111].

The average number of studying hours per day had a direct negative effect [β = -0.02, 95% CI: -0.04, -0.01] on depressive symptoms. This indicates that, as the number of study hours increases, scores for depressive symptoms decrease. This can be explained by the fact that when the number of study hours increases, students may have more confidence in their abilities and avoid worrying about taking tests. Which may improve their sleeping pattern, academic performance, and achievement [112]. In contrast to this finding, another study conducted in Iraq [58] showed that studying hours had a positive effect on depression. This discrepancy might be due to population differences. The previous study was conducted among health science students only. For health science students, there is a trend towards long time reading. Beyond that, the previous study used the Hospital Anxiety and Depression Scale (HADS-D), and it is known that it is considerably different from the PHQ-9 in measuring depressive symptoms [113].

A history of stressful life events had a direct positive effect [β = 0.07, 95% CI: 0.01, 0.13] on depressive symptoms. This indicates that having experienced stressful life events increases scores of depressive symptoms by 0.07, which is supported by a study conducted in Bangladesh [59]. This can be due to the fact that when students face stressful life events like the death of a family member or beloved one or the loss of a relationship, their jobs may cause sadness, or it might be related to the breakdown of the social support chain.

Problematic internet use had a direct positive effect [β = 0.23, 95% CI: 0.18, 0.27] on depression. Thus, when the PIU score increases, the level of depression also increases. Which is supported by other studies conducted in Ethiopia [97], Kenya [114], Iran [115], Spain [51], India [116], and Bangladesh [56]. This similarity might be due to the impact of long-term internet use on time management, which has a negative impact on studying and sleeping time [117,118]. To achieve their goals and responsibilities given to them by their parents, they may need to spend more time on their academic achievements. This complex relationship may lead to depressive symptoms.

Insomnia had a direct positive effect [β = 0.24, 95% CI: 0.17, 0.30] on depression. This can be explained as an increase in the score of insomnia increasing the level of depressive symptoms. Which is consistent with other studies conducted in Ethiopia [52] and Spain [51]. This can be explained by some theories, like that sleep loss can cause cognitive and mood changes, impairment in emotional regulation and stability, and alter neural processes, and that lack of sleep may induce a stress response and increase levels of inflammatory markers [119].

### Magnitude and determinants of ADHD

Our study revealed that the prevalence of ADHD was found to be 20.01% [95% CI: 18.1–22.1%]. This finding is in line with a study conducted among University students in Kenya [8], where the prevalence was explained as 21.8%. This similarity may be due to population and screening instrument similarities between the two studies. And the current finding is slightly lower than a study conducted among University students in Cameroon [9], where the prevalence was explained as 24.4%. This might be due to the population differences between the two studies, where the previous study was conducted among medical students alone.

Compared with other parts of the world, the current finding is much higher than the results of previous studies conducted in China [47,120,121], Korea [45], Iran [122,123], Belgium [124], and Turkey [7,44]. This discrepancy might be due to population differences; for instance, studies conducted in China and Belgium [47,123] were among medical students only; other studies conducted in China and Belgium were also among freshmen students [121,124], and a study conducted in Iran [122] included students within the age range of 17–35 years and MSc students. Another study conducted in Turkey excluded 5^th^ and 6^th^ year medicine students from the study [44]. This might be the effect of poor mental health literacy among non-health and freshmen students, which can result in poor mental health, and students who are prepared and able to adjust to the changes that moving into higher education presents also experience better mental health [125].

For studies conducted among Chinese and U.S. students [120] and in Turkey [7,44], the discrepancy might be due to differences in socio-demographic characteristics, methodology features, ethnic and cultural differences, and the diagnostic criteria involved in the studies [126]. However, a study conducted in Brazil [38] revealed a higher prevalence than the current study. This might be due to the fact that the previous study was conducted among medical students. Moreover, it is obvious that some of the increase in ADHD can be attributed to the development of the diagnostic criteria. Polanczyk and his colleagues have worked extensively on the epidemiology of ADHD, and they have consistently shown that variations in the measurement of outcomes, such as the diagnostic criteria for ADHD and the inclusion or exclusion of functional impairment, were largely responsible for the observed variability in prevalence rates across different studies [127,128].

#### Factors associated with ADHD

The year of study had a direct negative effect [β = -0.10, 95% CI: -0.12, -0.03] on ADHD. This indicates that when the year of study goes up by 1, the severity of ADHD decreases by 0.10, keeping other variables controlled. This explains that when students become familiar with the University environment, the symptoms may depreciate due to an increase in reduced interpersonal skills and coping with lifestyle and academic difficulties [129].

Our study also revealed that a mother’s education level had a direct negative effect [β = -0.09, 95% CI: -0.13, -0.06] on ADHD. This indicates that when the mother’s educational level increases by one, the severity of ADHD symptoms decreases by 0.09. This can be explained by the relationship between educational level and risky health behaviors during pregnancy. When the level of parental education increases, they may keep themselves from engaging in risky health behaviors since complications during delivery can cause ADHD. Thus, another theory that can be raised is the relationship between education and pregnancy-related complications; hence, education has a direct effect on reducing pregnancy-related complications [12,130]. Our finding is also supported by a previous study conducted in Sweden [42]. And studies conducted among children in Spain and Norway revealed that higher maternal and paternal educational attainment was associated with fewer symptoms of ADHD [131,132].

Birth order had a direct negative relationship [β = -0.09, 95% CI: -0.11, -0.07] with ADHD. This indicates that when birth order increases by 1, the severity of ADHD symptoms decreases by 0.09. This can also be explained by the relationship of ADHD with perinatal risk factors and birth-related complications, which have a huge contribution to ADHD. Thus, first-born babies may have faced higher pregnancy and obstetric-related complications compared to high-order babies. As evidenced by the literature, primiparous women are at high risk of these aforementioned complications [133]. As a result, children of primiparous mothers may have a higher risk of ADHD, which has the potential to persist into adulthood. This finding is similar to a previous study conducted among children and adolescents [134,135].

Chat chewing had a direct positive influence on ADHD [β = 0.18, 95% CI: 0.06, 0.30]. This implied that having ever used chat increases ADHD by 0.18. The effect of chat on ADHD can be explained by its relationship with neurobehavioral functioning. A recently conducted systematic review revealed that long-term use of chat was associated with significant deficits in several cognitive domains, including learning, motor speed and coordination, set-shifting and response inhibition functions, cognitive flexibility, short-term memory, and conflict resolution [136]. And a study conducted on animals (rats) highlights the adverse effects of chat on spatial learning and memory, which may have similar effects on humans too and specifically impair working memory [137].

Medically confirmed illness had a positive indirect effect [β = 0.14, 95% CI: 0.06, 0.22] on ADHD. This finding is congruent with a population-based study conducted in Sweden [20]. This might be due to the effect of ADHD on students’ lifestyles [101].

Another significant positive indirect effect on ADHD came from being worried about educational performance [β = 0.18, 95% CI: 0.13, 0.23]. This might be due to the presence of continuous worry among persons with ADHD; hence, they may occasionally act impulsively; they are generally introspective in their daily lives, and these thoughts cause worry. And also because of bad past experiences, the participants did not trust themselves in the future. In addition to this lack of daily routine, which manifested as having an irregular lifestyle and consistently breaking promises to themselves and others [129].

The average number of sleeping hours also had a protective indirect effect [β = -0.02, 95% CI: -0.03, -0.01] on adult ADHD symptoms. This can be explained by the fact that adequate sleeping time can reduce behavioral symptoms [138]. The current finding is also congruent with a study conducted among children showing that when sleeping hours decreased, the risk of ADHD increased [138]. In our study, the average number of studying hours also had a protective indirect effect [β = -0.02, 95% CI: -0.03, -0.01] on ADHD. This might be because when studying hours increase, students may build self-confidence and reduce the unwanted effect of ADHD on self-esteem. This may in turn influence their relationship with their colleagues, which can minimize their poor feelings about themselves [129].

A history of stressful life events had a positive indirect effect [β = 0.05, 95% CI: 0.003, 0.10] on ADHD. This finding is supported by a study conducted in Sweden [139]. This can be due to the fact that adults with ADHD may lose their relationships, their jobs, or their income [11]. Alcohol use had a positive indirect effect [β = 0.07, 95% CI: 0.03, 0.12] on ADHD, and this finding is supported by previous studies [44,46,47]. This can be explained as alcohol is a known depressant. Therefore, adults with ADHD may use alcohol to calm down their symptoms caused by hyperactivity. This might be the reason why alcohol use is indirectly related to ADHD.

Problematic internet use had a statistically significant indirect positive effect [β = 0.16, 95% CI: 0.13, 0.21] on ADHD through the mediational effect of depression, which is consistent with studies conducted in France [140] and Turkey [44]. This might be due to the effect of the impulsivity and hyperactivity of ADHD on their relationships with their friends and families, which can impose a feeling of loneliness, idleness, physical isolation, and a decrease in information and communication. Therefore, they may spend more time on the internet to gain pleasure and information.

Social support had an indirect protective effect [β = -0.16, 95% CI: -0.22, -0.12] on ADHD. This might be due to the fact that the presence of social support from friends or family may reduce depression. This statement is also supported by another finding [141]. Another study also explained that social support prevents the effect of ADHD on depression [142]. ADHD symptoms initially had a direct effect on depression, but it reduced to a non-significance effect after perceived social support was added. This may indicates lack of social support predominantly causes depression than it is caused by ADHD. This statement is supported by a longitudinal study conducted among adolescents aged 11–17 to test the social causation model to determine whether deficits in social support increase the likelihood of depression or interpersonal theories of depression (depression leads to social erosion), using a reciprocal influence model. The result indicated that depression resulted in social support erosion [143].

Insomnia had a statistically significant indirect positive effect [β = 0.17, 95% CI: 0.13, 0.23] on ADHD. This finding is consistent with a study conducted in Turkey [144]. This might be due to hyperactivity during the daytime, which may cause racing thoughts and a burst of energy at night that interfere with sleeping, or insomnia, which may worsen as people start to develop feelings of other psychiatric comorbidities like depression.

In the non-recursive relationship, depression had a significant effect on ADHD [β = 0.73, 95% CI: 0.62, 0.86]. This indicates that an increased depression score increases ADHD. The path from ADHD to depression had a negative effect [β = -0.14, 95% CI: -0.31, 0.02]. However, the estimate was not statistically significant (P-value = 0.09). This can be explained by the decline in total ADHD symptoms, especially hyperactivity [145]. The effects of childhood behavioral problems like emotionality may have an impact on their relationships with friends and families. Moreover, these bad past experiences may impose worry to the extent of affecting daily routines, which can be manifested by having an irregular lifestyle and consistently breaking promises to themselves and others. This symptom may predominate and become more apparent than previous symptoms of ADHD [129]. Moreover, Social support may prevent the effect of ADHD on depression [142].

## Strength and limitations of the study

Our study applied non-recursive SEM to assess the bidirectional relationship between ADHD and depression. In addition to this, compared to previous studies conducted in Ethiopia, the current study used a relatively large sample size to determine the magnitude of depression.

However, this study is not without limitations. We tested the causal relationship between outcome variables where the timelines were different, and our study included only university students, which may affect generalizability to all adults. In addition, we have utilized a self-administered questionnaire to study mental health problems; thus, participants may hide their past or current psychiatric history.

## Conclusion

The magnitude of ADHD and depression was found to be high. Factors like the presence of a medically confirmed chronic disease, a history of a head injury, a history of stressful life events, alcohol use, worrying about academic performance, the number of sleeping and studying hours, social support, PIU, and insomnia were significantly associated with depressive symptoms, while year of study, mother’s education level, birth order, khat chewing, and depressive symptoms were significantly related to ADHD. The presence of a medically confirmed chronic disease, worrying about academic performance, a history of stressful life events, alcohol use, a history of head injury, an average number of sleeping and studying hours, social support, PIU, and insomnia had a depression-mediated effect on ADHD.

However, the path from ADHD to depression and other variables including age, sex, cannabis use, current alcohol use, prior residence, marital status, presence of a friend, history of parental psychiatric problems, father education level, average monthly allowance, history of smoking, physical exercise, history of academic failure, and self-reported family economic level did not have a statistically significant direct and/or indirect relationship with either outcome variable.

## Recommendations

Our results have important implications for clinical and school-based prevention and intervention. Therefore, it is better that UoG work more on early screening and recognition of ADHD symptoms among students. That could partly serve as a preventive instrument for other comorbidities, like depression. Health sector managers should pay attention to improving the knowledge of the general population and health care professionals about these mental disorders. Furthermore, considering the high prevalence of ADHD and depression among students, timely diagnosis and treatment of these disorders are important. Moreover, there is a need to design appropriate interventions for university students. More studies are needed to determine the definite prevalence and determinant factors in the whole country. Thus, future researchers better to explore the relationship and magnitude of ADHD and depression longitudinally through interview-based methods and a diagnostician such as a psychiatrist.

## Supporting information

S1 Fig Modified measurement model with standardized estimates displayed, UoG, Northwest Ethiopia, 2022. (TIFF)

S2 Fig Modified measurement model after parceling with standardized estimates displayed, UoG, Northwest Ethiopia, 2022. (TIFF)

S1 Table The reliability and convergent validity of the constructs as measured by composite reliability and the average variance extracted, UoG, Northwest Ethiopia, 2022. (DOCX)

S2 Table Factor loadings for measurement component of latent variables during CFA of pilot study, UoG, Northwest Ethiopia, 2022. (DOCX)

S3 Table Response of the participants on each items of depression, UoG, Northwest Ethiopia, 2022 (n = 1504). (DOCX)

S4 Table Participant’s responses on each items of ADHD, UoG, Northwest Ethiopia, 2022. (DOCX)

S5 Table ICC result for each outcome variables, UoG, Northwest Ethiopia, 2022. (DOCX)

S6 Table Instrumental variable validity and strength test, UoG, Northwest Ethiopia, 2022. (DOCX)

S7 Table Modification indices among the error terms of the constructs, as covariance between each pair of items during CFA, UoG, Northwest Ethiopia, 2022. (DOCX)

S8 Table Parceling applied for three construct variables, UoG, Northwest Ethiopia, 2022. (DOCX)

## Abbreviations

ADHD: Attention Deficit Hyperactivity Disorder
AVE: Average Variance Extracted
CFA: Confirmatory Factor Analysis
PIU: Problematic Internet Use
SEM: Structural Equation Modeling
