# Supplementary figures and images for: Magnitude, relationship and determinants of attention deficit hyperactivity disorder and depression among University of Gondar undergraduate students, Northwest Ethiopia, 2022: Non-recursive structural equation modeling
Source: PLoS One. 2023 Oct 5;18(10):e0291137. doi: 10.1371/journal.pone.0291137 (PMC10553242; doi:10.1371/journal.pone.0291137)

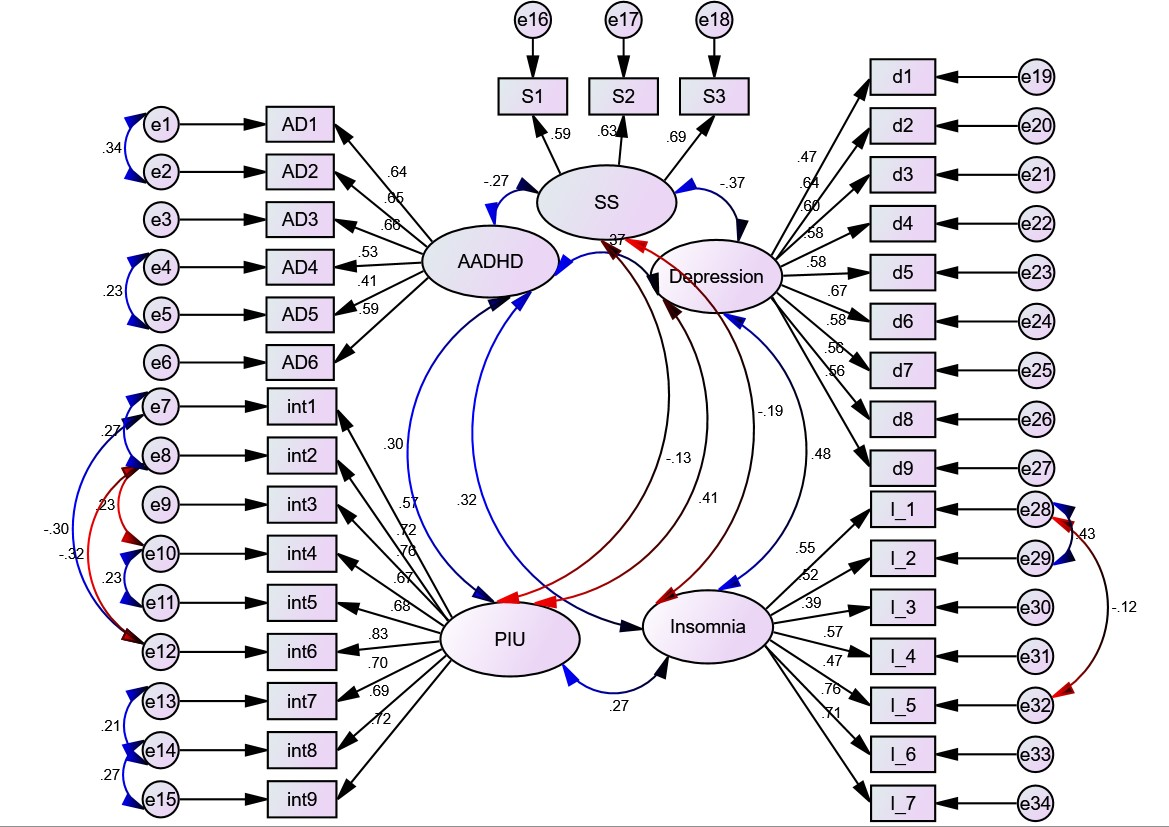

Supplement: S1 Fig — (TIFF) [file pone.0291137.s001.tiff]

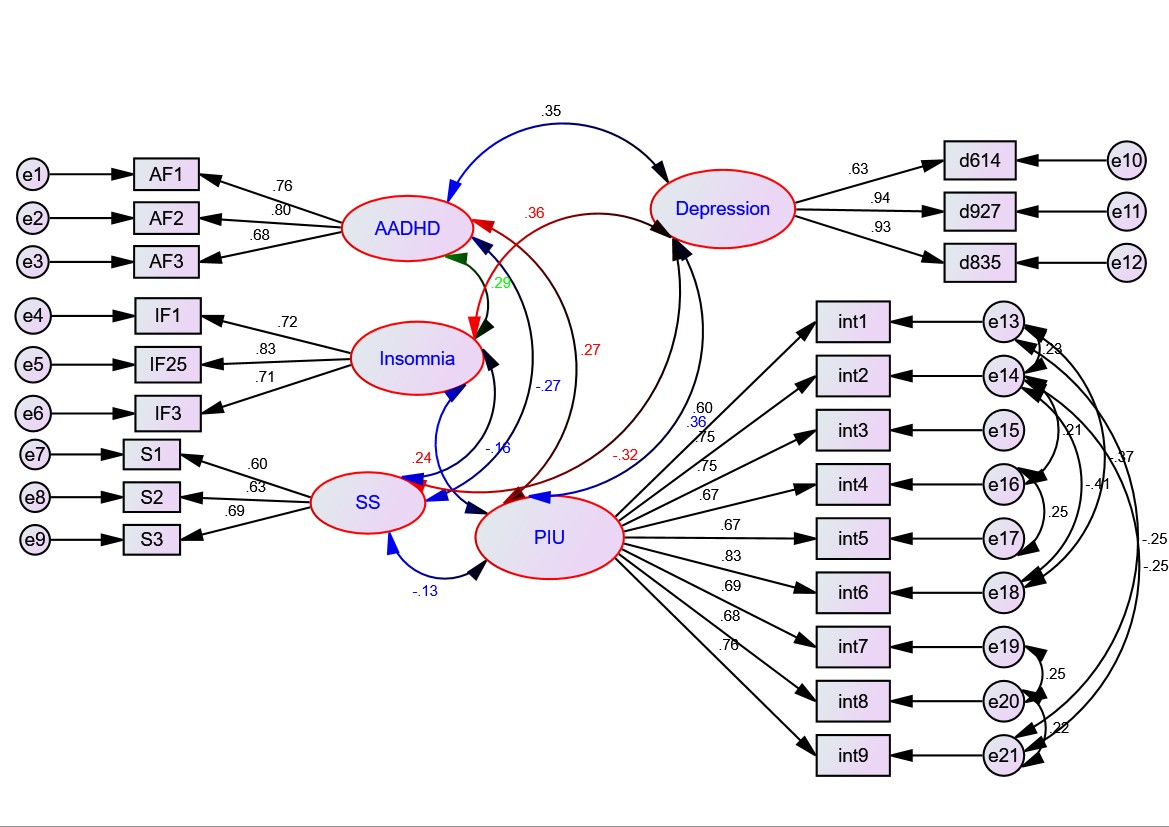

Supplement: S2 Fig — (TIFF) [file pone.0291137.s002.tiff]
