# Supplementary material for: Magnitude, relationship and determinants of attention deficit hyperactivity disorder and depression among University of Gondar undergraduate students, Northwest Ethiopia, 2022: Non-recursive structural equation modeling
Source: PLoS One. 2023 Oct 5;18(10):e0291137. doi: 10.1371/journal.pone.0291137 (PMC10553242; doi:10.1371/journal.pone.0291137)
Supplement: S1 Table — (DOCX) [file pone.0291137.s003.docx]

**S1 Table: The reliability and convergent validity of the constructs as measured by composite reliability and the average variance extracted, UoG, Northwest Ethiopia, 2022.**

| Construct | AVE | CR |
| --- | --- | --- |
| ADHD | 0.49 | 0.85 |
| Depression, | 0.46 | 0.88 |
| Insomnia | 0.47 | 0.86 |
| Social support | 0.38 | 0.64 |
| PIU | 0.40 | 0.86 |
