# Supplementary material for: Magnitude, relationship and determinants of attention deficit hyperactivity disorder and depression among University of Gondar undergraduate students, Northwest Ethiopia, 2022: Non-recursive structural equation modeling
Source: PLoS One. 2023 Oct 5;18(10):e0291137. doi: 10.1371/journal.pone.0291137 (PMC10553242; doi:10.1371/journal.pone.0291137)
Supplement: S2 Table — (DOCX) [file pone.0291137.s004.docx]

**S2 Table: Factor loadings for measurement component of latent variables during CFA of pilot study, UoG, Northwest Ethiopia, 2022.**

| Latent variables | Item | Standardized Coefficients | P – value |
| --- | --- | --- | --- |
| ADHD | ADHD1 | 0.63 | 0.000 |
|  | ADHD2 | 0.76 | 0.000 |
|  | ADHD3 | 0.67 | 0.000 |
|  | ADHD4 | 0.76 | 0.000 |
|  | ADHD5 | 0.72 | 0.000 |
|  | ADHD6 | 0.63 | 0.000 |
| Depression | D1 | 0.50 | 0.000 |
|  | D2 | 0.74 | 0.000 |
|  | D3 | 0.74 | 0.000 |
|  | D4 | 0.70 | 0.000 |
|  | D5 | 0.78 | 0.000 |
|  | D6 | 0.71 | 0.000 |
|  | D7 | 0.60 | 0.000 |
|  | D8 | 0.69 | 0.000 |
|  | D9 | 0.59 | 0.000 |
| Insomnia | I1 | 0.52 | 0.000 |
|  | I2 | 0.54 | 0.000 |
|  | I3 | 0.65 | 0.000 |
|  | I4 | 0.71 | 0.000 |
|  | I5 | 0.68 | 0.000 |
|  | I6 | 0.87 | 0.000 |
|  | I7 | 0.76 | 0.000 |
| Social support | S1 | 0.62 | 0.000 |
|  | S2 | 0.72 | 0.000 |
|  | S3 | 0.50 | 0.000 |
| PIU | INT1 | 0.52 | 0.000 |
|  | INT2 | 0.71 | 0.000 |
|  | INT3 | 0.63 | 0.000 |
|  | INT4 | 0.65 | 0.000 |
|  | INT5 | 0.71 | 0.000 |
|  | INT6 | 0.64 | 0.000 |
|  | INT7 | 0.69 | 0.000 |
|  | INT8 | 0.60 | 0.000 |
|  | INT9 | 0.54 | 0.000 |
