# Supplementary material for: Magnitude, relationship and determinants of attention deficit hyperactivity disorder and depression among University of Gondar undergraduate students, Northwest Ethiopia, 2022: Non-recursive structural equation modeling
Source: PLoS One. 2023 Oct 5;18(10):e0291137. doi: 10.1371/journal.pone.0291137 (PMC10553242; doi:10.1371/journal.pone.0291137)
Supplement: S3 Table — (DOCX) [file pone.0291137.s005.docx]

**S3 Table: Response of the participants on each items of depression, UoG, Northwest Ethiopia, 2022 (n=1504)**

| Depression | Responses | | | |  |
| --- | --- | --- | --- | --- | --- |
| Items | Not at all(0) | Several days(1) | More than half of the days(2) | Every day (3) | Total |
| Depression 1 | 477(31.7%) | 433(28.8%) | 389(25.9%) | 205(13.6%) | 1504 |
| Depression 2 | 581(38.6%) | 424(28.2%) | 366(24.3%) | 133(8.8%) | 1504 |
| Depression 3 | 592(39.4%) | 409(27.2%) | 331(22%) | 172(11.4%) | 1504 |
| Depression 4 | 492(32.7%) | 467(31.1%) | 386(25.7%) | 159(10.6%) | 1504 |
| Depression 5 | 673(44.8%) | 383(25.5%) | 336(22.3%) | 112(7.5%) | 1504 |
| Depression 6 | 851(56.6%) | 308(20.5%) | 223(14.8%) | 122(8.1%) | 1504 |
| Depression 7 | 434(28.9%) | 493(32.8%) | 362(24.1%) | 215(14.3%) | 1504 |
| Depression 8 | 805(53.5%) | 347(23.1%) | 259(17.2%) | 93(6.2%) | 1504 |
| Depression 9 | 1108(73.7%) | 187(12.4%) | 145(9.6%) | 64(4.3%) | 1504 |
