# Supplementary material for: Magnitude, relationship and determinants of attention deficit hyperactivity disorder and depression among University of Gondar undergraduate students, Northwest Ethiopia, 2022: Non-recursive structural equation modeling
Source: PLoS One. 2023 Oct 5;18(10):e0291137. doi: 10.1371/journal.pone.0291137 (PMC10553242; doi:10.1371/journal.pone.0291137)
Supplement: S4 Table — (DOCX) [file pone.0291137.s006.docx]

**S4 Table: Participant’s responses on each items of ADHD, UoG, Northwest Ethiopia, 2022.**

| ADHD | Response | | | | | Total |
| --- | --- | --- | --- | --- | --- | --- |
| Item | Never (0) | Rarely (1) | Sometimes (2) | Often (3) | Very often (4) | 1504 |
| ADHD 1 | 136(9%) | 476(31.6%) | 487(32.4%) | 207(13.8%) | 198(13.2%) | 1504 |
| ADHD 2 | 152(10.1%) | 520(34.6%) | 411(27.3%) | 234(15.6%) | 187(12.4%) | 1504 |
| ADHD 3 | 235(15.6%) | 516(34.3%) | 363(24.1%) | 215(14.3%) | 175(11.6%) | 1504 |
| ADHD 4 | 153(10.2%) | 330(21.9%) | 526(35%) | 313(20.8%) | 182(12.1%) | 1504 |
| ADHD 5 | 138(9.2%) | 279(18.6%) | 468(31.1%) | 315(20.9%) | 304(20.2%) | 1504 |
| ADHD 6 | 238(15.8%) | 345(22.9%) | 591(39.3%) | 206(13.7%) | 124(8.2%) | 1504 |
