# Supplementary material for: Magnitude, relationship and determinants of attention deficit hyperactivity disorder and depression among University of Gondar undergraduate students, Northwest Ethiopia, 2022: Non-recursive structural equation modeling
Source: PLoS One. 2023 Oct 5;18(10):e0291137. doi: 10.1371/journal.pone.0291137 (PMC10553242; doi:10.1371/journal.pone.0291137)
Supplement: S5 Table — (DOCX) [file pone.0291137.s007.docx]

**S5 Table: ICC result for each outcome variables, UoG, Northwest Ethiopia, 2022.**

| Outcome variables | Clustering variable | ICC [95% CI] |
| --- | --- | --- |
| Adult ADHD | College | 0.03 [0.01, 0.08] |
|  | Department | 0.03 [0.01, 0.08] |
| Depression | College | 0.01 [0.002, 0.06] |
|  | Department | 0.01 [0.01. 0.05] |
