# Supplementary material for: Magnitude, relationship and determinants of attention deficit hyperactivity disorder and depression among University of Gondar undergraduate students, Northwest Ethiopia, 2022: Non-recursive structural equation modeling
Source: PLoS One. 2023 Oct 5;18(10):e0291137. doi: 10.1371/journal.pone.0291137 (PMC10553242; doi:10.1371/journal.pone.0291137)
Supplement: S6 Table — (DOCX) [file pone.0291137.s008.docx]

**S6 Table: Instrumental variable validity and strength test, UoG, Northwest Ethiopia, 2022.**

| Outcome variable | Instrumental variables | Instrumented | Anderson canon. corr. LM(under identification test), Chi-sq (P-value) | Cragg-Donald Wald F statistic (weak identification test) | Sargan statistic (over identification test of all instruments), Chi-sq (P-value) |
| --- | --- | --- | --- | --- | --- |
| ADHD | Birth order, mother and father education level | Depression | 88.13, 3 (0.00) | 30.846 | 0.773, 2(0.68) |
| Depression | Social support, insomnia, PIU, stressful life event and worry about academic performance | Adult ADHD | 386.6, 5(0.00) | 102.567 | 2.1, 4(0.72) |
