# Supplementary material for: Magnitude, relationship and determinants of attention deficit hyperactivity disorder and depression among University of Gondar undergraduate students, Northwest Ethiopia, 2022: Non-recursive structural equation modeling
Source: PLoS One. 2023 Oct 5;18(10):e0291137. doi: 10.1371/journal.pone.0291137 (PMC10553242; doi:10.1371/journal.pone.0291137)
Supplement: S7 Table — (DOCX) [file pone.0291137.s009.docx]

**S7 Table: Modification indices among the error terms of the constructs, as covariance between each pair of items during CFA, UoG, Northwest Ethiopia, 2022.**

| Construct | Errors correlated | MI | SEPC |
| --- | --- | --- | --- |
| ADHD | e1 <--> e2 | 61.03 | 0.16 |
|  | e4<--> e5 | 92.70 | 0.29 |
| PIU | e8 <--> e7 | 182.4 | 0.36 |
|  | e10 <--> e8 | 146.68 | 0.27 |
|  | e10<--> e11 | 57.24 | 0.17 |
|  | e12 <--> e7 | 74.63 | 0.21 |
|  | e12 <--> e8 | 82.34 | 0.18 |
|  | e14<--> e13 | 61.09 | 0.17 |
|  | e15 <--> e14 | 97.74 | 0.20 |
| Insomnia | e28<--> e29 | 219.44 | 0.28 |
|  | e28 <--> e32 | 44.83 | 0.17 |
