# Supplementary material for: Magnitude, relationship and determinants of attention deficit hyperactivity disorder and depression among University of Gondar undergraduate students, Northwest Ethiopia, 2022: Non-recursive structural equation modeling
Source: PLoS One. 2023 Oct 5;18(10):e0291137. doi: 10.1371/journal.pone.0291137 (PMC10553242; doi:10.1371/journal.pone.0291137)
Supplement: S8 Table — (DOCX) [file pone.0291137.s010.docx]

**S8 Table: Parceling applied for three construct variables, UoG, Northwest Ethiopia, 2022.**

| Construct | Parcel | Items |
| --- | --- | --- |
| ADHD | Parcel 1 | AD2 and AD5 |
|  | Parcel 2 | AD1 and AD4 |
|  | Parcel 3 | AD3 and AD6 |
| Depression | Parcel 1 | d6, d1 and d4 |
|  | Parcel 2 | d9, d2 and d7 |
|  | Parcel 3 | d8, d3 and d5 |
| Insomnia | Parcel 1 | I_3 and I_6 |
|  | Parcel 2 | I_5, I_7 and I_2 |
|  | Parcel 3 | I_4 and I_1 |
